# Metabolic Signatures for Liver Cancer Diagnosis and Mechanistic Insights: A Large‐Scale, Multicenter Study

**DOI:** 10.1002/advs.78077

**Published:** 2026-10-04

**Authors:** Yongjie Xu, Jiachen Wang, Chunmeng Ding, Changfa Xia, Yunyong Liu, Yuanjie Zheng, Sheng Chang, Shaokai Zhang, Yutong He, Ruifang Sun, Yunfeng Xi, Yan Liu, Kun Qian, Wanqing Chen

**Affiliations:** ^1^ National Cancer Center/National Clinical Research Center for Cancer/Cancer Hospital Chinese Academy of Medical Sciences and Peking Union Medical College Beijing China; ^2^ School of Biomedical Engineering, Institute of Medical Robotics and Shanghai Academy of Experimental Medicine Shanghai Jiao Tong University Shanghai China; ^3^ National Cancer Center/National Clinical Research Center for Cancer/Cancer Hospital & Shenzhen Hospital Chinese Academy of Medical Sciences and Peking Union Medical College Guangdong China; ^4^ Department of Cancer Epidemiology, Affiliated Cancer Hospital of Zhengzhou University and Henan Cancer Hospital, Henan Engineering Research Center of Cancer Prevention and Control Henan International Joint Laboratory of Cancer Prevention Zhengzhou China; ^5^ Cancer Institute the Fourth Hospital of Hebei Medical University/the Tumor Hospital of Hebei Province Hebei China; ^6^ Department of Tumor Biobank, Shanxi Province Cancer Hospital, Shanxi Hospital Affiliated to Cancer Hospital, Chinese Academy of Medical Sciences Cancer Hospital Affiliated to Shanxi Medical University Shanxi China; ^7^ The Inner Mongolia Autonomous Region Center for Disease Control and Prevention Inner Mongolia China; ^8^ Senior Department of Infectious Diseases/National Clinical Research Center for Infectious Diseases Chinese PLA General Hospital Beijing China

**Keywords:** early diagnosis, HIF1α, liver cancer, metabolites, nicotinamide

## Abstract

The poor prognosis of liver cancer (LC) highlights the urgent need for more effective strategies for early detection. We conducted a large, multicenter study to develop and validate a serum metabolic signature for LC diagnosis. Using high‐throughput nanoparticle‐enhanced laser desorption/ionization mass spectrometry, we profiled serum metabolites from 1,924 participants in discovery cohort recruited across 11 clinical centers and 225 participants in independent external validation cohort from two additional centers. A nine‐metabolite signature combined with alpha‐fetoprotein achieved excellent discriminatory performance, with an AUC of 0.92 in the discovery cohort and 0.93 in the external validation cohort. The combined model also showed good sensitivity for early‐stage LC, reaching 0.85 and 0.78 in the two cohorts, respectively, and performed particularly well for hepatocellular carcinoma, with AUCs of 0.94 and 0.93. Exploratory analyses suggested potential utility for intrahepatic cholangiocarcinoma diagnosis. Mendelian randomization analysis supported a potential causal association between nicotinamide (NAM) levels and LC risk. Functional studies showed that NAM promotes LC cell proliferation, migration, and invasion, through an NAD^+^/SIRT1‐dependent mechanism that increases HIF1α stability and activates glycolytic and MAPK signaling. Overall, we developed a promising metabolic diagnostic signature for LC and identified NAM‐associated metabolic signaling as a potential contributor to LC progression.

## Graphical abstract

A large‐scale multicenter serum metabolomics study (13 centers, n = 2,149) identified a 9‐metabolite + AFP diagnostic signature for liver cancer (AUC = 0.93). Among the signature, nicotinamide (NAM) was validated as an oncometabolite: NAM enhances NAD^+^ synthesis, activating SIRT1 to deacetylate and stabilize HIF1α, thereby driving glycolysis, MAPK signaling, and liver cancer progression. (Created by BioRender. Agreement number: MX2A30XD5R).

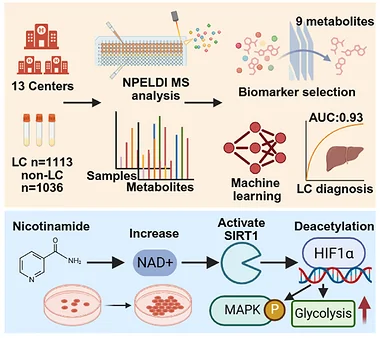

## 1 Introduction

Liver cancer (LC) is the sixth most common cancer and the third leading cause of cancer‐related mortality globally [1, 2]. The high mortality rate is largely attributable to its poor prognosis, with a five‐year survival rate of only 15.1% [3]. Hepatocellular carcinoma (HCC) accounts for the majority of cases (75%–85%), while intrahepatic cholangiocarcinoma (ICC) comprises a smaller subset (10%–15%) [4, 5].

Early detection is the cornerstone of improving outcomes for LC patients. The current surveillance standard, which combines alpha‐fetoprotein (AFP) testing with abdominal ultrasound, has been shown to improve HCC survival in our recent cohort study [6]. Nonetheless, the sensitivity of AFP for HCC remains limited (approximately 45.5%), and even when combined with ultrasound, sensitivity fails to exceed 65% [6]. This significant diagnostic gap severely curtails the potential benefits of screening programs and highlights an urgent, unmet clinical need for novel and more effective biomarkers.

Metabolomics has emerged as a powerful approach for biomarker discovery, offering direct insight into the functional endpoints of pathological processes [7]. Previous investigations have identified various metabolites and panels, such as phenylalanine‐tryptophan, methionine, glycocholic acid, and bile acids, with potential diagnostic value for LC [8, 9, 10, 11, 12, 13]. However, these studies were consistently constrained by several key limitations: a predominant focus on HCC to the exclusion of ICC; reliance on small, single‐center cohorts that lacked geographical diversity and were thus susceptible to confounding by regional dietary and lifestyle factors; and a frequent absence of rigorous, independent external validation. Furthermore, conventional analytical platforms like liquid chromatography‐mass spectrometry (LC‐MS) often face challenges related to throughput and reproducibility.

To directly address these critical gaps, we conducted a large‐scale, multicenter study comprising more than 2,000 samples from 13 medical centers across diverse geographic regions in China. Using nanoparticle‐enhanced laser desorption/ionization mass spectrometry (NPELDI‐MS), a high‐throughput platform, we developed and validated a metabolic signature for early detection of LC, with robust performance in HCC and preliminary evidence of potential utility in ICC. Mechanistically, we revealed that nicotinamide (NAM) promotes LC progression by activating glycolysis and MAPK signaling through the NAD^+^/SIRT1/HIF1α axis. Our findings underscore the potential to integrate metabolic profiling into clinical practice, facilitating early diagnosis and metabolic intervention in patients with LC.

## 2 Results

### 2.1 Demographics of the Study Cohort

The participant flowchart is presented in Figure 1. The discovery cohort retrospectively enrolled 1,924 subjects from 11 centers of five provinces, comprising 1,013 LC patients and 911 non‐LC controls. The median age was 58.00 years in the LC group and 54.00 years in the control group. Males constituted 79.37% of LC cases and 52.47% of non‐LC cases. HBsAg positivity was observed in 75.02% of LC patients and 73.66% of controls. AFP levels were significantly elevated in the LC group compared to the non‐LC group (Figure S1). HCC was the predominant pathological type (n = 833), followed by ICC (n = 107).

**FIGURE 1:**
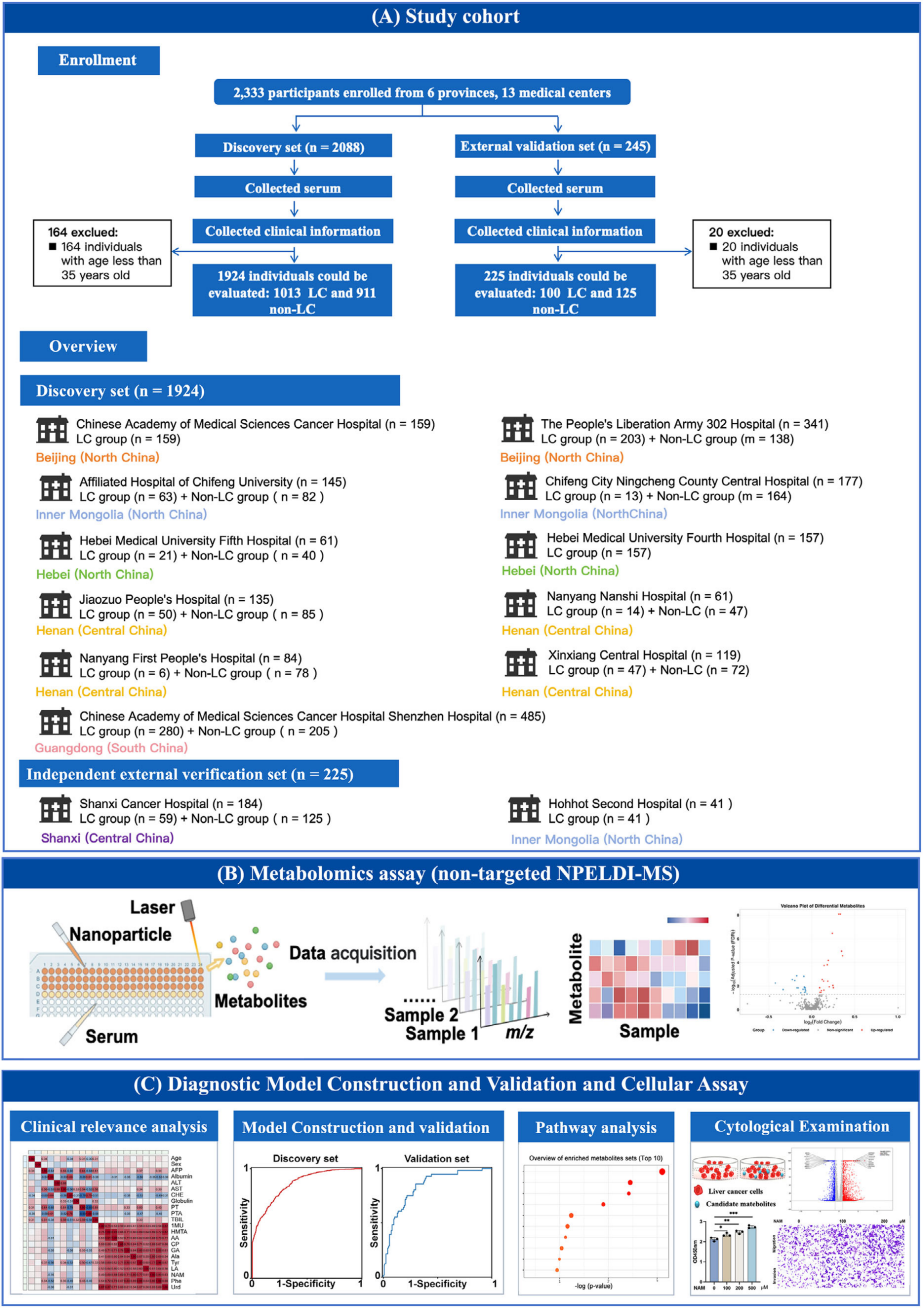
Study workflow for serum metabolomic profiling in LC. (A) Enrollment and overview of study cohort. (B) Microarrayed non‐targeted NPELDI‐MS was employed to detect differential metabolites. (C) LC diagnostic model construction, optimization, and validation, followed by functional examination of key metabolites in vitro. LC, liver cancer. NPELDI‐MS, nanoparticle‐enhanced laser desorption/ionisation mass spectrometry.

The external validation cohort recruited 225 subjects from two centers, including 100 LC patients and 125 non‐LC controls. The median age was 62.00 years in the LC group and 60.00 years in the non‐LC group. Males accounted for 85.00% of LC cases and 68.00% of non‐LC controls. HBsAg positivity was present in 69 (69.00%) LC patients and 43 (34.40%) controls. Consistent with the discovery cohort, AFP levels were markedly higher in LC patients than in controls (Figure S1). HCC remained the dominant pathological subtype (n = 84), followed by ICC (n = 15). Detailed demographic and clinical characteristics of both cohorts are summarized in Table 1.

**TABLE 1:** Clinical characteristics of the liver cancer and non‐liver cancer participants.

	Discovery set			External validation set		
	LC (n = 1013)	Non‐LC (n = 911)	P value	LC (n = 100)	Non‐LC (n = 125)	P value
Age (years), median (P25, P75)						
	58.00 (51.00, 66.00)	54.00 (45.00, 63.00)	<0.001	62.00 (56.00, 67.00)	60.00 (50.00, 71.00)	0.66
Sex, n(%)						
Male	804 (79.37)	478 (52.47)	<0.001	85 (85.00)	85 (68.00)	0.01
Female	209 (20.63)	433 (47.53)		15 (15.00)	40 (32.00)	
HBsAg, n(%)						
Positive	760 (75.02)	671 (73.66)	0.53	69 (69.00)	43 (34.40)	<0.001
Negative	253 (24.98)	240 (26.34)		31 (31.00)	82 (65.60)	
Stage, n(%)						
I	317 (31.29)	—		37 (37.00)	—	
II	118 (11.65)	—		22 (22.00)	—	
III	218 (21.52)	—		23 (23.00)	—	
IV	243 (23.99)	—		14 (14.00)	—	
Unknown	117 (11.55)	—		4 (4.00)	—	
Pathological type, n(%)						
HCC	833 (82.23)	—		84 (84.00)	—	
ICC	107 (10.56)	—		15 (15.00)	—	
Others	5 (0.50)	—		1 (1.00)	—	
Unknown	68 (6.71)	—		0 (0)	—	
Cirrhosis, n(%)						
Yes		92 (10.10)			16 (12.80)	
No/unknown		819 (89.90)			109 (87.20)	
AFP (ng/mL), median (P25, P75)						
	8.06 (2.78, 126.29)	1.71 (1.09, 2.61)	<0.001	11.01 (2.73, 75.22)	1.09 (0.76, 1.98)	<0.001

LC, liver cancer; HCC, hepatocellular carcinoma; ICC, intrahepatic cholangiocarcinoma

### 2.2 High‐Throughput Serum Metabolic Profiling Using NPELDI‐MS

We established a rapid NPELDI‐MS platform capable of analyzing serum metabolites within seconds per sample using minimal sample volume (1 µL of 10‐fold diluted serum) without requiring prior enrichment or separation. This high‐throughput approach generated spectra with total ion counts of approximately 2.06×10^7^, showing prominent metabolic features in the low mass range (100–500 Da, Figure 2A). After preprocessing, we identified 340 reproducible metabolic features (Figure 2B).

**FIGURE 2:**
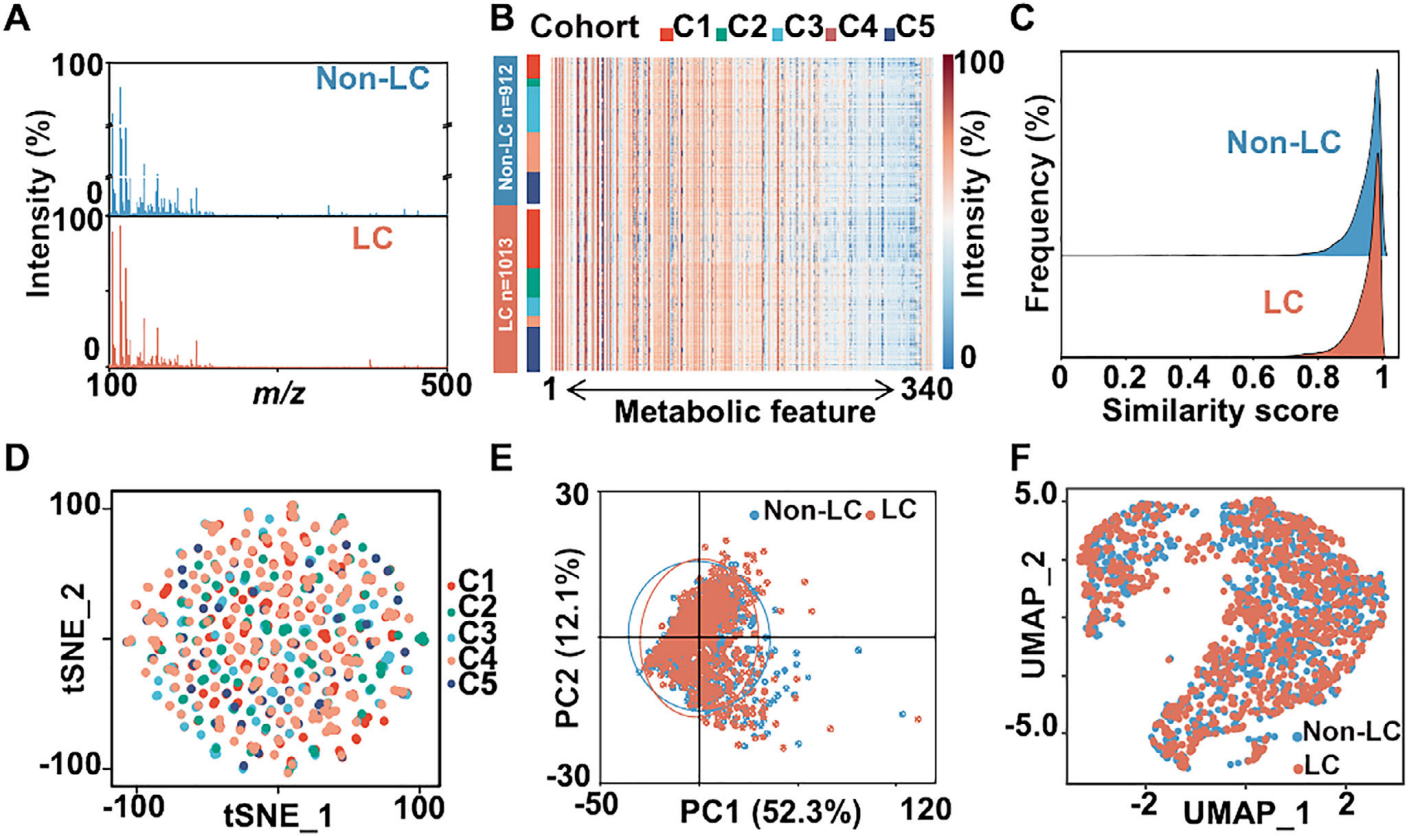
Metabolic features in a comparative cohort. (A) Representative mass spectra derived from non‐targeted NPELDI‐MS analysis within LC and non‐LC groups individually. (B) Clustered heatmap of 340 metabolic features in all samples. (C) Frequency distribution of similarity scores within LC and non‐LC groups. (D) The tSNE result of LC samples from five centers exhibited robustness of cohort inclusion. (E) PCA and (F) UMAP visualized the metabolic differences between LC and non‐LC, which need to be further explored. C1, Beijing cohort; C2, Hebei cohort; C3, Henan cohort; C4, Inner Mongolia cohort; C5, Guangdong cohort. LC, liver cancer. NPELDI‐MS, nanoparticle‐enhanced laser desorption/ionization mass spectrometry. PCA, principal component analysis. tSNE, t‐distributed stochastic neighbor embedding. UMAP, uniform manifold approximation and projection.

Quality assessment demonstrated excellent technical reproducibility, with intra‐group cosine similarity scores exceeding 0.9 in over 85% of comparisons (Figure 2C). To further evaluate the reproducibility and stability of the method, we analyzed quality control (QC) samples using principal component analysis (PCA) and calculated the relative standard deviations (RSD) of selected metabolite intensities (Figure S2). All 48 QC tests clustered tightly in the PCA score plot, indicating minimal instrumental variation throughout the entire detection process. Moreover, the RSD values of five representative metabolite intensities across all QC samples ranged from 19.5% to 22.6%, all falling below the widely accepted threshold of 30% for metabolomics studies [14]. These results confirm that the NPELDI‐MS platform exhibited satisfactory analytical reproducibility and stability during the entire sample detection workflow.

We further explored the sample distribution patterns using dimensionality reduction analyses, stratified by study center and by clinical subgroup. T‐distributed stochastic neighbor embedding (t‐SNE) visualization revealed no center‐specific clustering (Figure 2D), supporting the integration of multi‐center data. Unsupervised methods, PCA and uniform manifold approximation and projection (UMAP), showed substantial overlap between LC and non‐LC groups (Figure 2E,F). This observation highlighted the inherent limitation of unsupervised dimensionality reduction in high‐dimensional settings, where biologically meaningful signals can be masked by global variance structure. Supervised machine learning approaches that explicitly model the relationship between features and group labels are better suited for extracting distinguishing signatures in complex datasets.

### 2.3 Differential Metabolite Discovery and Clinical Correlations

Initial screening identified 37 potentially significant features with a false discovery rate (FDR) < 0.05 from the 340 detected metabolic features (Figure 3A, Table S1), which were annotated to 31 metabolites—16 upregulated and 15 downregulated in LC patients (Table S2). A Naïve Bayes model constructed with all 31 metabolites achieved an AUC of 0.83 (95% CI: 0.82‐0.85) with an accuracy of 73.7% in distinguishing LC from non‐LC individuals (Figure 3B). To improve clinical translatability and model simplicity, we applied LASSO regression to refine the metabolite panel, yielding an optimized 11‐metabolite (11‐M) panel comprising features with importance scores ≥ 0.20 (Figure 3C, Figure S3, Table S2). The model was evaluated using 5‐fold cross‐validation in the discovery cohort to mitigate overfitting. After adjusting for age, gender, and study center, the refined 11‐M panel achieved an AUC of 0.85 (95% CI: 0.83‐0.86) with an accuracy of 75.4% (Figure 3D), confirming robust diagnostic performance.

**FIGURE 3:**
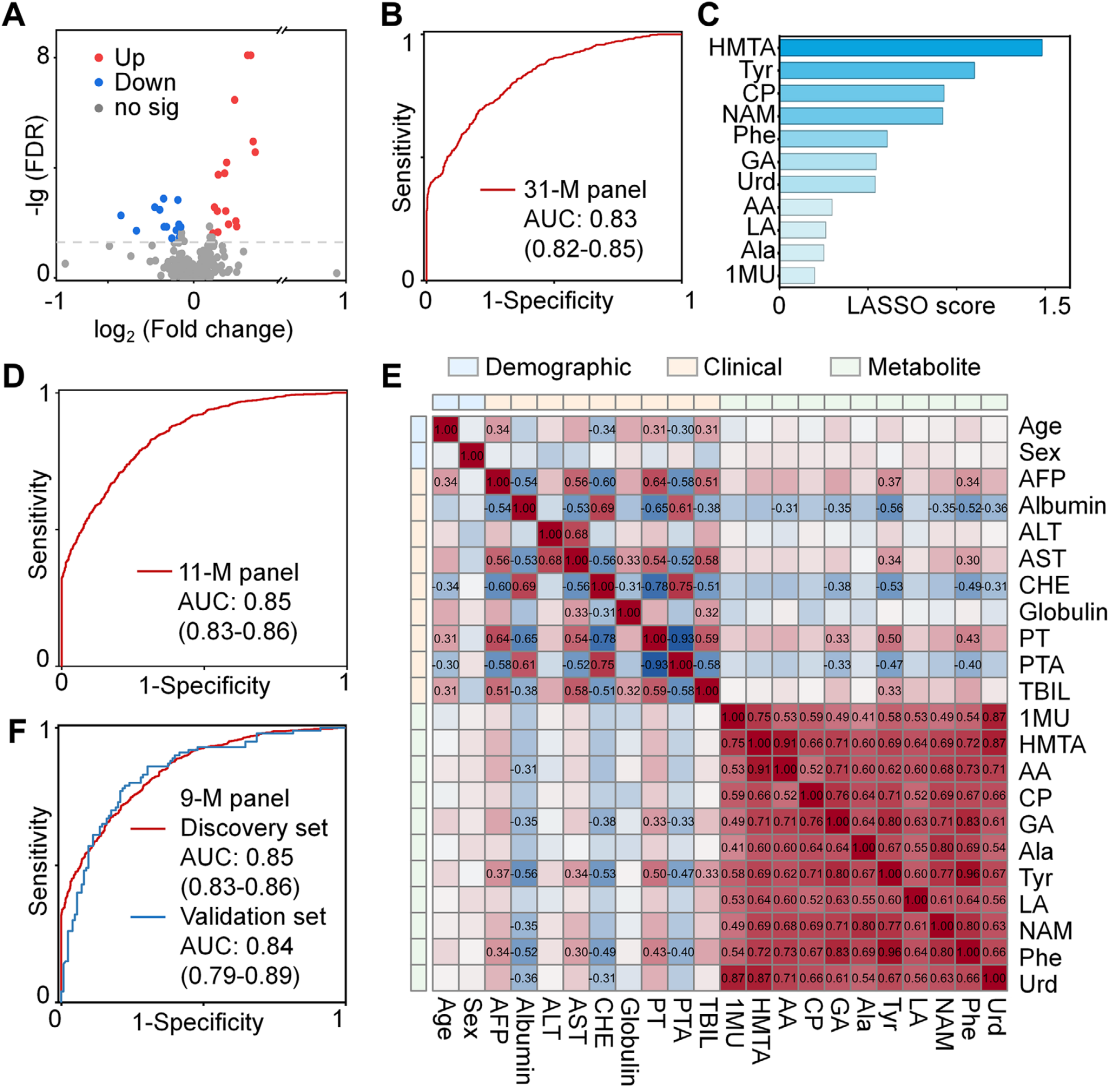
Differential metabolite profiling and model development in LC. (A) Volcano plot illustrating 340 metabolic features in LC patients compared with non‐LC controls, showing significantly upregulated signals (red), downregulated signals (blue), and non‐significant signals (gray). (B) Naïve Bayes model was constructed using 31 differential metabolites (31‐M) for the diagnosis of LC. (C) Ranking of 31 differential metabolites based on LASSO coefficients. 11 Metabolites with LASSO coefficients ≥ 0.20 were exhibited and selected for model construction. (D) Naïve Bayes model constructed using 11 differential metabolites (11‐M) with 5‐fold cross‐validation. (E) Spearman correlation matrix among demographic indicators, clinical indicators, and 11 selected metabolites. Only correlations with |r|>0.3 and FDR<0.05 are shown. (F) ROC curve for the construction (red) and validation (blue) of a 9‐M based Naïve Bayes model in LC diagnosis. AFP, alpha‐fetoprotein. ALT, alanine aminotransferase. AST, aspartate transaminase. CHE, cholinesterase. PT, prothrombin time. PTA, prothrombin activity. TBIL, total bilirubin. 1MU, 1‐Methyluric acid. HMTA, 2‐Hydroxy‐3‐methylbutyric acid. AA, Acetoacetic acid. CP, Carbamoyl phosphate. GA, Glyoxylic acid. Ala, alanine. Tyr, tyrosine. LA, Linoleic acid. NAM, nicotinamide. Phe, Phenylalanine. Urd, Uridine. LC, liver cancer. ROC, receiver operating characteristic.

Analysis of the 11 metabolites with clinical parameters revealed significant associations between key metabolites and liver function indicators. Phenylalanine and tyrosine levels showed positive correlations with prothrombin time (PT) and aspartate transaminase (AST), and negative correlations with prothrombin activity (PTA), cholinesterase (CHE), and albumin, a pattern indicative of impaired hepatic synthetic function and cellular injury (Figure 3E). The significant positive correlation between these aromatic amino acids and AFP levels further suggests a potential link to tumor aggressiveness. Furthermore, we observed significant negative correlations between serum albumin, a robust surrogate for functional hepatic mass and synthetic reserve, and other panel metabolites, including acetoacetic acid, glyoxylic acid, NAM, and uridine. This pattern underscores that the dysregulated metabolic signature captured by our panel is deeply embedded within the context of progressive hepatic dysfunction, a hallmark of advanced LC.

### 2.4 LC Diagnostic Model Construction, Optimization, and Validation

To further enhance model parsimony and clinical translatability, we refined the initial metabolite panel. Given the exceptionally high correlation between phenylalanine and tyrosine (r = 0.96), and between 2‐hydroxy‐3‐methylbutyric acid and acetoacetic acid (r = 0.91, Figure 3E), we hypothesized these pairs conveyed redundant diagnostic information. By removing phenylalanine and acetoacetic acid from each pair, we derived a streamlined 9‐metabolite (9‐M) panel, comprising uridine, NAM, L‐alanine, L‐tyrosine, glyoxylic acid, carbamoyl phosphate, linoleic acid, 1‐methyluric acid, and 2‐hydroxy‐3‐methylbutyric acid. This optimized panel retained diagnostic performance comparable to the original 11‐M set, achieving AUC values of 0.85 (95% CI: 0.83‐0.86; accuracy: 74.8%) in the discovery cohort and 0.84 (95% CI: 0.79‐0.89; accuracy: 72.4%) in the external validation cohort (Figure 3F). Crucially, the 9‐M panel demonstrated robust discriminatory capacity in the clinically challenging subgroup of AFP‐negative individuals in both cohorts (Figure 4A), addressing a key limitation of current screening strategies.

**FIGURE 4:**
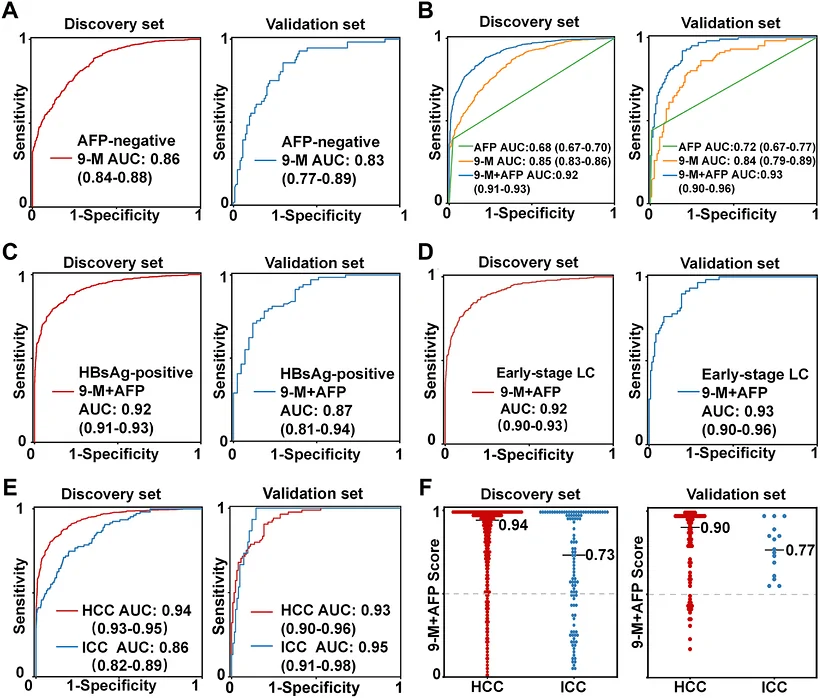
Construction and validation of LC diagnostic models. (A) ROC curve for the construction and validation of the 9‐M based Naïve Bayes model in LC diagnosis among the AFP‐negative population. (B) ROC curves for the construction and validation of diagnostic performance in LC: AFP (green), Naïve Bayes model built with 9 metabolites (9‐M, orange), and combined Naïve Bayes model integrating 9 metabolites with AFP (9‐M+AFP, blue). (C) ROC curve of 9‐M+AFP Naïve Bayes model in LC diagnosis among the hepatitis B surface antigen (HBsAg)‐positive population. (D) ROC curve of 9‐M+AFP Naïve Bayes model in the diagnosis of early‐stage LC (Stage I & II). (E and F) ROC curve (E) and model score (F) of 9‐M+AFP Naïve Bayes model in the diagnosis of HCC (red) and ICC (blue). The horizontal bars in (F) indicate the median of each group. LC, liver cancer. HCC, hepatocellular carcinoma. ICC, intrahepatic cholangiocarcinoma. ROC, receiver operating characteristic.

Integration of the 9‐M panel with serum AFP (combined model: 9‐M+AFP) markedly enhanced diagnostic accuracy. The combined model achieved excellent AUC values of 0.92 (95% CI: 0.91–0.93; accuracy: 83.6%) in the discovery set and 0.93 (95% CI: 0.90‐0.96; accuracy: 84.0%) in the validation set (Figure 4B), representing a significant improvement over AFP alone. Notably, the combined model dramatically increased sensitivity from 0.39 (95%CI: 0.36‐0.42) to 0.84 (95% CI: 0.82‐0.86) in the discovery cohort and from 0.43 (95%CI: 0.34‐0.53) to 0.83 (95% CI: 0.74‐0.89) in the validation cohort, while maintaining relatively high specificity (Table S3). This model also exhibited excellent clinical utility by effectively distinguishing hepatitis B virus (HBV)‐related LC from chronic hepatitis B (CHB), a major high‐risk population targeted for LC surveillance (Figure 4C). In addition, the model effectively distinguished LC from cirrhosis, another key population requiring regular surveillance due to its substantially elevated risk of malignant transformation (Figure S4A). Furthermore, it maintained high performance for detecting early‐stage (Stage I & II) LC, with sensitivities of 0.85 (95% CI: 0.82‐0.88) in discovery cohort and 0.78 (95% CI: 0.66‐0.87) in validation cohort (Figure 4D, Table S3). For AFP‑negative early‑stage LC patients, the model also demonstrated strong diagnostic performance (Figure S4B). Subgroup analysis by pathological type revealed that the model achieved high accuracy for HCC, with AUCs of 0.94 (95% CI: 0.93–0.95, accuracy: 85.5%) in the discovery cohort and 0.93 (95% CI: 0.90–0.96, accuracy: 84.5%) in the external validation cohort. Encouraging discriminative capacity was also observed for ICC, with AUCs of 0.86 (95% CI: 0.82–0.89, accuracy: 82.9%) in the discovery cohort and 0.95 (95% CI: 0.91–0.98, accuracy: 86.4%) in the validation cohort (Figure 4E,F). Decision curve analysis further confirmed the clinical net benefit of the combined model (Figure S4C).

### 2.5 NAM Promotes LC Cell Proliferation, Migration, and Invasion

Accumulating evidence underscores that metabolites not only reflect disease phenotypes but also actively shape biological functions [15]. To explore the functional roles of dysregulated metabolites in LC, we performed pathway enrichment analysis on the 31 differential metabolites. This analysis revealed their primary involvement in amino acid metabolism pathways, such as aromatic amino acid biosynthesis and metabolism, and alanine, aspartate, and glutamate metabolism (Figure 5A), pathways well‐established to contribute to hepatocarcinogenesis [16, 17, 18, 19]. Intriguingly, we also identified significant enrichment in the nicotinate and nicotinamide metabolism pathway. Metabolites belonging to the vitamin B3 family were upregulated in LC (Table S2), suggesting a potential link between vitamin B3 metabolic dysregulation and LC development.

**FIGURE 5:**
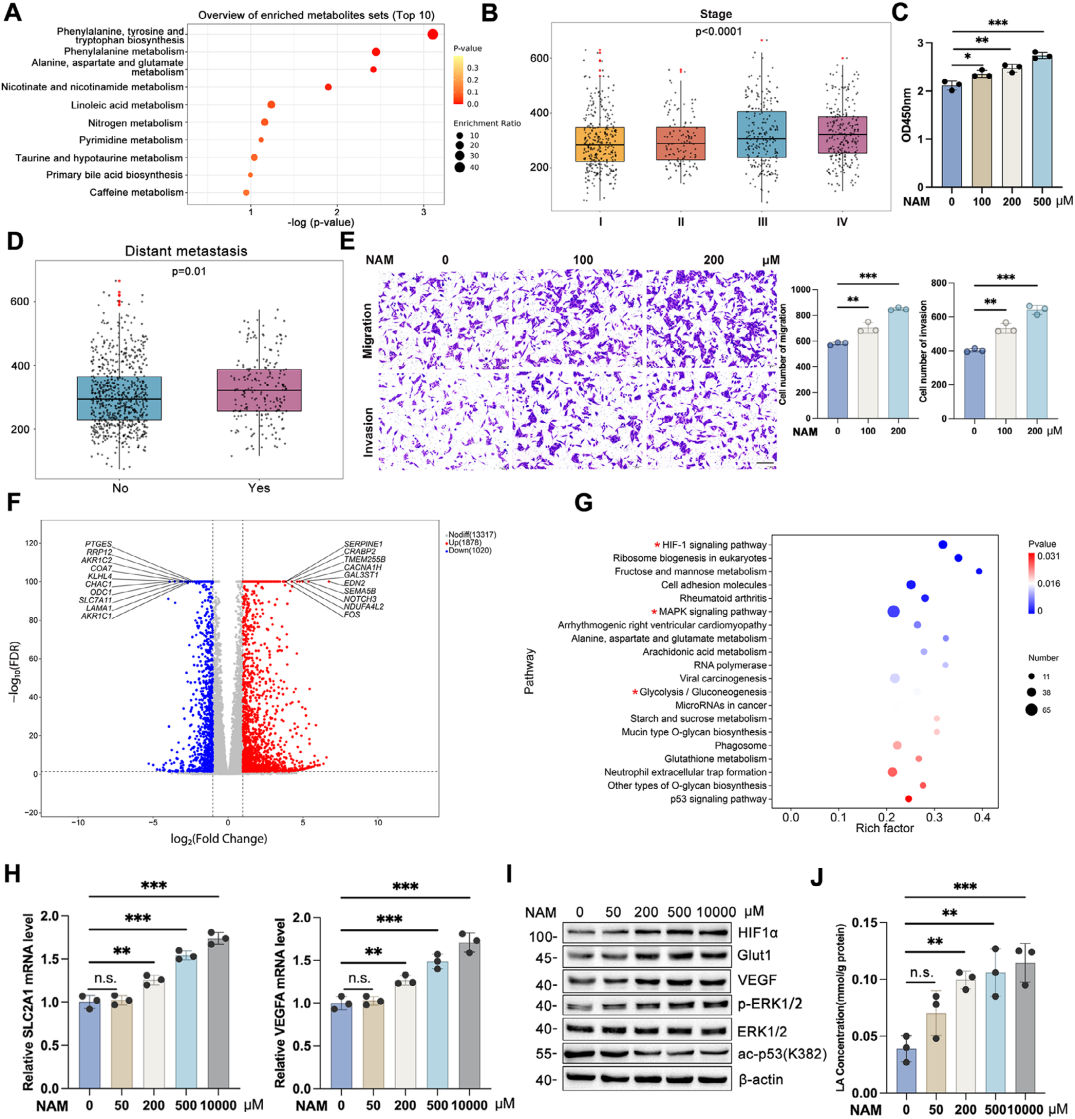
NAM promotes LC cell proliferation, migration, and invasion. (A) KEGG enrichment analysis of 31 dysregulated metabolites in LC. (B) Box plots showing the differences in NAM levels in LC patients from Stage I‐IV, adjusted for age, gender, HBsAg status, and location. Statistical analysis was performed using analysis of covariance, with the overall adjusted p‐value displayed. (C) Cell viability of HepG2 cells treated with the indicated concentrations of NAM for 72 h. (D) Box plots showing the differences in NAM levels between LC patients with distant metastasis and those without distant metastasis, adjusted for age, gender, HBsAg status, and location. Statistical analysis was performed using the Wilcoxon rank‐sum test with covariate adjustment. (E) Representative images and quantification of migration and invasion assays for HepG2 cells treated with the indicated concentrations of NAM. Scale bar, 200 µm. (F) Volcano plot displaying significantly up‑regulated (red) and down‑regulated (blue) genes in HepG2 cells treated with 200 µM NAM (fold change ≥ 2, FDR < 0.05). (G) KEGG enrichment analysis of differentially expressed genes from (F), highlighting significant enrichment in the HIF1α, MAPK, and glycolysis pathways. (H) mRNA expression levels of SLC2A1 (encoding Glut1) and VEGFA (encoding VEGF) in HepG2 cells treated with increasing concentrations of NAM. (I) Western blot analysis of protein expression in HepG2 cells following treatment with different concentrations of NAM. (J) Intracellular LA production in HepG2 cells after treatment with various concentrations of NAM. LA, lactate. LC, liver cancer. NAM, nicotinamide. ^*^p<0.05, ^**^p<0.01, ^***^p<0.001, ^****^p<0.0001. n.s., not significant.

Focusing on NAM, Mendelian randomization analysis supported a potential causal association between genetically predicted NAM levels and LC risk (Figure S5A, Table S4). Clinically, serum NAM levels showed a progressive increase with advancing tumor stage (Figure 5B). In vitro functional assays confirmed that NAM promotes LC cell proliferation in a concentration‐dependent manner (Figure 5C, Figure S5B). Notably, patients with distant metastasis exhibited significantly higher serum NAM concentrations than those without metastasis (Figure 5D). This clinical association was functionally validated in vitro, where NAM treatment robustly enhanced the migratory and invasive capacities of LC cells (Figure 5E, Figure S5C), indicating that NAM actively promotes LC progression and metastasis.

To elucidate the underlying mechanisms, we performed RNA sequencing on NAM‐treated LC cells, which identified 1,878 upregulated and 1,020 downregulated genes (Figure 5F). Pathway enrichment analysis of these 2,898 differentially expressed genes demonstrated significant activation of the HIF1α signaling pathway, mitogen‐activated protein kinase (MAPK) pathway, and glycolysis, all established drivers of cancer progression (Figure 5G). Subsequent molecular validation confirmed that NAM upregulates HIF1α protein expression and activates its downstream MAPK and glycolytic pathways (Figure 5H–J, Figure S5D–F), providing a mechanistic foundation for its tumor‐promoting effects in LC.

### 2.6 NAM Upregulates HIF1α Expression via the NAD+/SIRT1‐Dependent Pathway

To elucidate the underlying mechanism by which NAM promotes LC progression, we first focused on its role in nicotinamide adenine dinucleotide (NAD^+^) metabolism, given that NAM is a key precursor for intracellular NAD^+^ biosynthesis. Consistent with this, the pro‐proliferative effect of NAM on LC cells was completely abrogated by treatment with Daporinad, a specific inhibitor of nicotinamide phosphoribosyltransferase (NAMPT, the rate‐limiting enzyme in the NAD^+^ salvage pathway, Figure 6A). Further validating our hypothesis, NAM treatment significantly elevated intracellular NAD^+^ levels and increased the NAD^+^/NADH ratio in LC cells (Figure S6A), confirming that NAM exerts its oncogenic effects, at least in part, through enhancing NAD^+^ synthesis.

**FIGURE 6:**
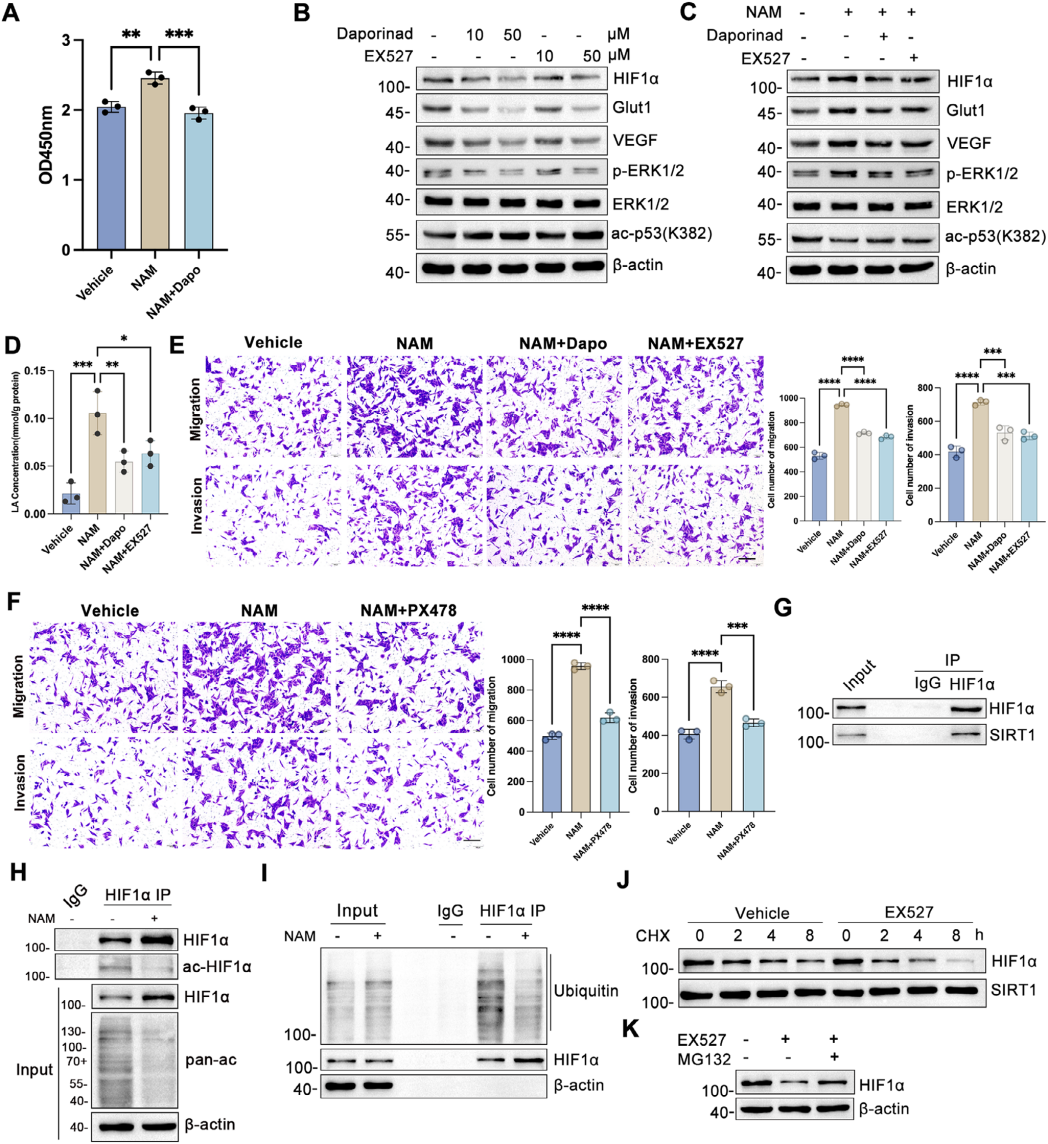
NAM upregulates HIF1α expression via an NAD^+^/SIRT1‐dependent pathway. (A) Cell viability of HepG2 cells treated with 200 µM NAM, with or without co‐treatment with 50 µM Daporinad (NAMPT inhibitor). (B) Western blot analysis of protein expression in HepG2 cells following treatment with different concentrations of Daporinad or EX527 (SIRT1 inhibitor). (C) Western blot analysis of protein expression in HepG2 cells following treatment with 200 µM NAM with or without 50 µM Daporinad and 50 µM EX527. (D) Intracellular LA production in HepG2 cells after treatment with 200 µM NAM with or without co‐treatment with 50 µM Daporinad and 50 µM EX527. (E) Representative images and quantification of migration and invasion assays for HepG2 cells treated with 200 µM NAM with or without co‐treatment with 50 µM Daporinad and 50 µM EX527. Scale bar, 200 µm. (F) Representative images and quantification of migration and invasion assays for HepG2 cells treated with 200 µM NAM with or without co‐treatment with 20 µM PX478 (HIF1α inhibitor). Scale bar, 200 µm. (G) HepG2 cells were treated with 200 µM NAM for 24 h, followed by co‐IP with an anti‑HIF1α antibody to detect the interaction between HIF1α and SIRT1. (H) HepG2 cells were incubated with 200 µM NAM or vehicle for 24 h. Cells were then harvested, and total protein was extracted for co‐immunoprecipitation assays to detect the acetylation level of HIF1α. (I) HepG2 cells were treated with 200 µM NAM or vehicle for 24 h, with MG132 (20 µM) added 4 h before harvest. Cell lysates were subjected to co‑IP to detect the ubiquitination level of HIF1α. (J) HepG2 cells were pretreated with 200 µM NAM for 24 h to elevate basal HIF1α levels, then treated with 10 µg/mL CHX for the indicated time points, with or without 50 µM EX527 pretreatment. HIF1α protein levels were assessed by immunoblotting. Quantification of HIF1α protein decay is shown in Figure S6B. (K) Immunoblot analysis of HIF1α expression in HepG2 cells pretreated with 200 µM NAM for 24 h, followed by treatment with 50 µM EX527 for 24 h in the presence or absence of the proteasome inhibitor MG132 (20 µM, added 4 h before harvest). Co‐IP, co‑immunoprecipitation. CHX, cycloheximide. LA, lactate. NAM, nicotinamide. *p<0.05, ^**^p<0.01, ^***^p<0.001, ^****^p<0.0001.

Notably, NAD^+^ serves not only as a metabolic cofactor supporting cell proliferation but also as an essential activator of sirtuin deacetylases (SIRTs), a family of NAD^+^‐dependent enzymes. We therefore postulated that the NAM/NAD^+^‐mediated upregulation of HIF1α might be dependent on SIRT1, the most well‐characterized member of the sirtuin family. To test this, we treated LC cells with either Daporinad (to block NAD^+^ synthesis) or EX527 (a selective SIRT1 inhibitor). Pharmacological inhibition of either NAD^+^ synthesis or SIRT1 activity effectively suppressed HIF1α protein expression and downregulated the transcription of its downstream target genes (Figure 6B). The inhibitory effect of EX527 on SIRT1 activity was confirmed by assessing the acetylation levels of p53 (a classical SIRT1 substrate) at Lys382 (Figure 5I and Figure 6B). Critically, the NAM‐induced upregulation of HIF1α and the subsequent activation of its downstream signaling cascades were fully reversed by co‐treatment with either Daporinad or EX527 (Figure 6C,D). Moreover, functional assays demonstrated that NAM‐driven enhancements in LC cell migration and invasion were abrogated when the NAD^+^/SIRT1/HIF1α axis was inhibited (Figure 6E,F), highlighting the functional relevance of this pathway in NAM‐mediated LC progression.

To further dissect the molecular mechanism by which SIRT1 regulates HIF1α, we focused on HIF1α acetylation status, as previous studies have established that acetylation of HIF1α promotes its proteasomal degradation [20]. We therefore hypothesized that SIRT1 stabilizes HIF1α through deacetylation. In support of this hypothesis, co‐immunoprecipitation (co‐IP) assays confirmed a physical interaction between HIF1α and SIRT1 (Figure 6G), and NAM treatment significantly reduced the acetylation level of HIF1α in LC cells (Figure 6H), suggesting that SIRT1‐mediated deacetylation is a key event in NAM‐induced HIF1α stabilization.

To further validate this mechanism, we assessed the ubiquitination status of HIF1α following NAM treatment. Notably, NAM treatment markedly decreased the ubiquitination level of HIF1α (Figure 6I), indicating that SIRT1‐mediated deacetylation protects HIF1α from ubiquitin‐proteasome‐dependent degradation. Consistent with this finding, cycloheximide chase assays revealed that EX527 significantly shortened the half‐life of HIF1α, and the EX527‐induced reduction in HIF1α levels was rescued by co‐treatment with the proteasome inhibitor MG132 (Figure 6J,K, Figure S6B).

Collectively, these findings demonstrate that NAM activates the NAD^+^/SIRT1 signaling axis, which in turn deacetylates and stabilizes HIF1α through a proteasome‐dependent mechanism. This stabilization leads to the accumulation of HIF1α in LC cells, followed by activation of downstream pathways including MAPK signaling and glycolysis. Together, these events collectively drive LC progression by promoting cell proliferation, migration, and invasion.

### 2.7 NAM is Associated With HBV‐Related LC

Given the established role of SIRT1 in enhancing the replication of HBV and promoting HBV‐related LC tumorigenesis [21, 22, 23], and our finding that NAM activates SIRT1 in LC cells, we sought to determine whether NAM is specifically associated with HBV‐driven LC. We measured serum NAM levels across four distinct cohorts in the discovery sets: healthy controls (HC), patients with CHB, patients with non‐HBV‐related LC (non‐HBV‐LC), and patients with HBV‐related LC (HBV‐LC).

Serum NAM levels were significantly elevated in the CHB group compared with HC group. Notably, NAM levels in the HBV‑LC group were also markedly higher than those in the non‑HBV‑LC group (Figure 7A). Importantly, patients with HBV‑LC exhibited significantly higher NAM concentrations than patients with CHB, whereas no significant difference was observed between non‑HBV‑LC patients and HC. This stepwise pattern of NAM accumulation, HC < CHB < HBV‐LC, with non‐HBV‐LC remaining at baseline, strongly suggests that NAM elevation is specifically linked to HBV‐associated malignant progression, rather than being a general feature of all LC subtypes. To further support a direct link between serum NAM levels and HBV viral activity, we analyzed a Beijing sub‐cohort consisting of 112 treatment‐naïve HBV‐LC patients. In this sub‐cohort, we observed a significant positive correlation between serum NAM levels and serum HBV DNA copy number (Figure 7B), indicating that NAM levels are closely associated with HBV replication status in HBV‐LC patients.

**FIGURE 7:**
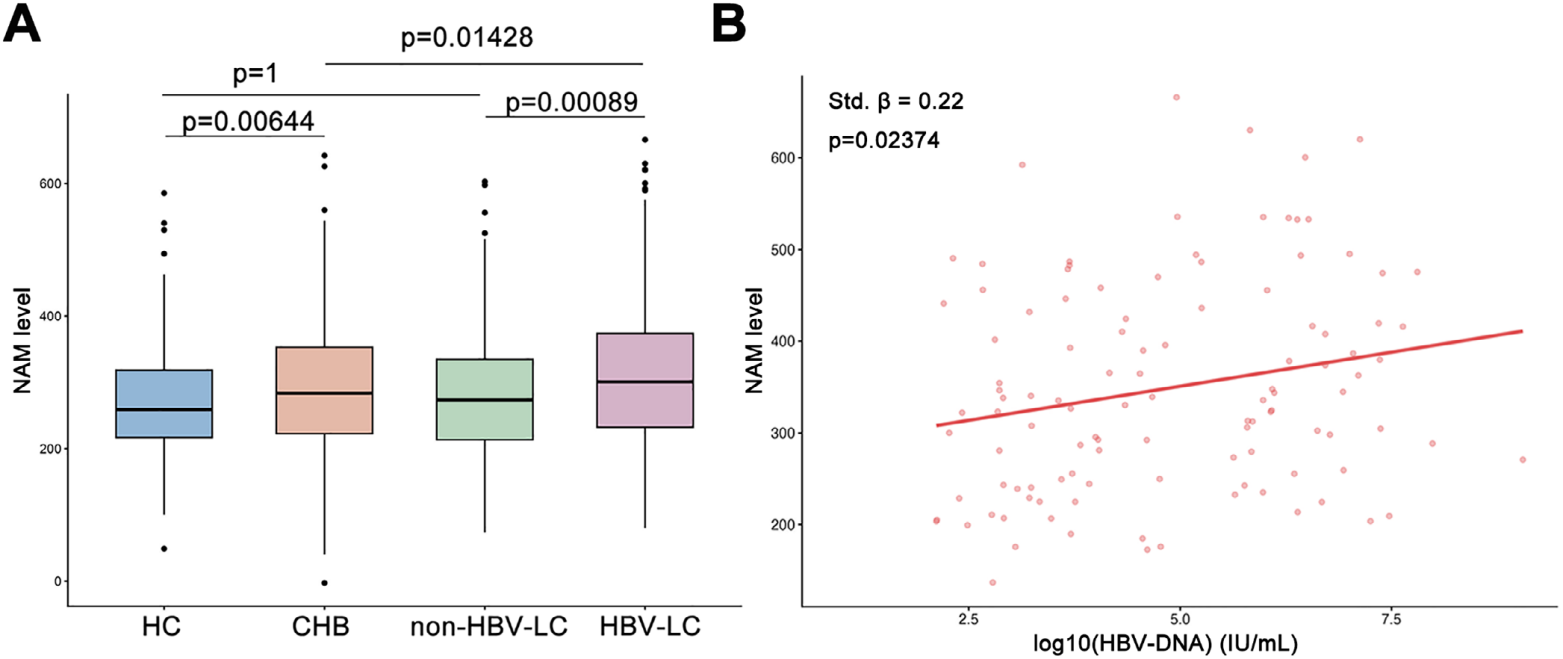
NAM is associated with hepatitis B virus‐related LC. (A) The four groups include HC, CHB, non‐HBV‐LC, and HBV‐LC, with a statistically significant overall difference in serum NAM levels among four groups (Kruskal‐Wallis rank sum test, p < 0.001). Pairwise comparisons were performed using Wilcoxon rank‐sum test with Bonferroni correction for multiple testing adjustments. (B) Scatter plot showing the positive correlation between log10‐transformed HBV DNA levels (IU/mL) and serum NAM concentration in 112 HBV‐LC patients. The analysis was adjusted for age and sex using a multiple linear regression model. HC, healthy controls. CHB, chronic hepatitis B patients. non‐HBV‐LC, non‐hepatitis B‐related liver cancer patients. HBV‐LC, hepatitis B‐related LC patients. NAM, nicotinamide.

## 3 Discussion

The persistently poor prognosis of LC underscores the critical importance of effective screening and early detection for improving patient survival. However, the suboptimal sensitivity of AFP, the most widely used serological biomarker for LC screening, severely limits its benefit and has delayed the establishment of a definitive mortality reduction from screening programs. Consequently, the discovery of novel biomarkers to augment current diagnostic approaches is essential for enhancing the efficiency and effectiveness of LC screening.

Alternative biomarkers, including AFP‐L3 and des‐gamma‐carboxy prothrombin (DCP), demonstrate high specificity but are hampered by inconsistent and frequently inadequate sensitivity, particularly for early‐stage LC [24, 25, 26]. Composite models such as the GALAD score, which incorporates gender, age, AFP‐L3, AFP, and DCP, aim to leverage the complementary strengths of these markers [27, 28]. However, they often achieve only marginal gains in sensitivity at the expense of increased false‐positive rates, failing to provide a definitive solution [26].

In this context, metabolomics presents a compelling alternative. Positioned at the terminus of the central dogma, the metabolome acts as a magnifying glass for physiological states, where subtle changes in upstream genes or proteins are amplified into significant metabolic perturbations [7]. Metabolites are remarkably stable in biofluids, require minimal sample volume, and are less demanding in terms of sample quality, making them ideal candidates for biomarker discovery [29]. This potential has been validated in multicenter studies for other cancers, such as lung cancer and colorectal cancer [30, 31]. Our study leverages a transformative analytical technology, NPELDI‐MS, which overcomes key limitations of traditional methods like LC‐MS by requiring minimal sample pretreatment (minutes vs. hours) and consuming a minuscule 1 µL of serum per test. Its ultra‐high throughput, analyzing over 300 samples per chip, unlocks the potential for large‐scale biomarker validation. Crucially, we have recently presented a diagnostic method coupling dried serum spots and NPELDI‐MS, creating a standardized, end‐to‐end pipeline from sample collection and ambient‐temperature transport to centralized laboratory analysis [32]. This innovation dramatically reduces logistical barriers and costs, making high‐quality metabolic screening feasible even in underserved regions, thereby addressing the critical issue of accessibility.

A principal challenge in metabolomic biomarker development has been the limited generalizability of models beyond their derivation cohorts. As the central organ for metabolism, the liver's metabolite profile is profoundly influenced by regional dietary patterns and environmental factors, often resulting in models that excel in single‐center studies but falter during external, multicenter validation. Consequently, the identification of metabolites that are intrinsically linked to disease progression rather than confounded by lifestyle or geographic variation is critical for building robust and generalizable diagnostic models. Our study was specifically designed to address this challenge. By enrolling a large, geographically diverse cohort from 11 centers across China, and rigorously validating our model in an independent cohort including two centers, we have established a metabolic signature resilient to these confounders. The combined 9‐M+AFP model achieved an AUC exceeding 0.90 in external validation for early‐stage LC, underscoring its exceptional and transferable performance.

Crucially, the nine metabolites constituting our diagnostic panel are not merely statistical features; most of them are grounded in the established biology of hepatocarcinogenesis. For example, phenylalanine/tyrosine metabolism has been extensively confirmed to be associated with the occurrence and development of LC [16, 17, 18]. Linoleic acid, previously shown to suppress HCC cell proliferation [33], was downregulated in our model. Carbamoyl phosphate, whose synthesis is governed by the widely downregulated enzyme Carbamoyl Phosphate Synthetase 1 in LC patients [19, 34], was also a key component. 2‐hydroxy‐3‐methylbutyric acid, a branched‐chain amino acid (BCAA) metabolite, was found at reduced levels, aligning with the known loss of BCAA catabolism during cancer progression [35]. 1‐methyluric acid, a major metabolite from tea and coffee (beverages with established protective effects against LC) [36, 37, 38, 39], was also integral to our panel. This biological grounding significantly enhances the model's extrapolative potential, as it captures conserved core pathways of liver carcinogenesis, even if background metabolic baselines vary across populations.

Of particular interest is our finding that NAM, a well‐known agent for skin cancer prevention [40], is elevated in the serum of LC patients and appears to function as a key driver of LC progression. Both Mendelian randomization analysis and in vitro functional assays suggested its tumor‐promoting role in LC. Mechanistically, we demonstrated that NAM upregulates HIF1α expression and activates downstream MAPK and glycolysis pathways in an NAD^+^/SIRT1‐dependent manner.

It should be noted that the NAM concentrations used in our in vitro experiments may not exactly match those present within the tumor microenvironment. Although the concentrations adopted here are widely used in comparable mechanistic studies [15, 31, 33], they cannot fully recapitulate the local NAM levels that cancer cells encounter in vivo. Direct quantification of NAM within the tumor microenvironment remains technically challenging. Serum NAM mainly reflects systemic NAM availability rather than the local tumor milieu, whereas bulk tissue measurements yield average values across heterogeneous cell populations and fail to capture the NAM concentration immediately adjacent to cancer cells. Despite these limitations, we observed pro‐tumorigenic effects of NAM on LC cell proliferation, migration and invasion across multiple concentrations. Activation of the NAD^+^/SIRT1/HIF1α axis was concentration‐dependent but not confined to a narrow concentration range. This indicates that NAM's pro‐tumorigenic activity is a general property of this pathway, rather than an effect occurring only at a specific concentration. Furthermore, our clinical data demonstrate elevated serum NAM in LC patients, correlated with advanced stage and distant metastasis, supporting the in vivo relevance of this signaling axis. We recognize that direct measurement of intratumoral NAM would provide more precise reference values. Future work utilizing spatially resolved metabolomics or paired tumor interstitial fluid analysis may better define the local NAM concentrations experienced by cancer cells.

These results appear to contradict numerous studies describing NAM as an inhibitor of SIRT1, largely based on its feedback inhibition of sirtuin activity [41]. However, our data indicate that in LC cells, NAM predominantly enhances SIRT1 activity. We propose that this discrepancy stems from the cellular metabolic context: NAM serves as a precursor for NAD^+^ biosynthesis, and elevated intracellular NAD^+^ levels are a prerequisite for SIRT1 activation. While NAM may transiently inhibit SIRT1 in cell‐free systems or shortly after cellular treatment due to direct enzymatic feedback, its sustained effect in cells is the promotion of NAD^+^ synthesis and consequent SIRT1 activation over time. This interpretation is consistent with the work of Hwang et al. [41], who reported that although NAM acutely inhibits SIRT1 in vitro, where it cannot be converted to NAD^+^ via the lack of NAMPT, it ultimately activates SIRT1 in cellular settings. In our experiments, SIRT1 activity was enhanced even at high exogenous NAM concentrations (up to 10 mM), further supporting the view that within the physiological and pathophysiological range in LC cells, NAM functions primarily as a SIRT1 activator rather than an inhibitor.

Furthermore, we found that the tumor‐promoting effect of NAM is subtype‐specific, being particularly prominent in HBV‐related LC. Given that serum NAM levels were significantly higher in patients with chronic HBV infection compared to non‐infected individuals, we hypothesize that HBV infection elevates systemic NAM, which in turn activates SIRT1. This establishes a pathogenic positive feedback loop: SIRT1 activation further enhances HBV replication [21, 22, 23], leading to additional NAM accumulation, while simultaneously promoting LC progression through upregulation of MAPK signaling and glycolysis, the latter being strongly associated with development, progression, and poor prognosis of LC [5, 42]. This mechanism carries important clinical implications. First, our findings raise the hypothesis that excessive NAM supplementation may pose a risk in HBV‐related LC, which warrants careful evaluation in future clinical and epidemiological studies. Second, for HBV‐related LC patients with elevated serum NAM, therapeutic targeting of NAD^+^ biosynthesis, for example, using NAMPT inhibitors, may represent a promising strategy, although further validation in animal models and clinical trials is warranted.

Our work also advances the field beyond previous foundational studies. For example, while Luo et al. [8] identified a diagnostic model performing well for early HCC detection, its efficacy for ICC was limited. In contrast, our 9‐M+AFP panel demonstrated robust diagnostic performance in HCC and also showed encouraging discriminative capacity in ICC in exploratory subgroup analyses. Although HCC and ICC differ in etiology, risk factors, and screening strategies, they share a common downstream diagnostic pathway in clinical practice: screen‐positive individuals typically proceed to contrast‐enhanced magnetic resonance imaging or biopsy for definitive diagnosis. Therefore, even when the model is primarily applied for HCC screening, incidentally detected ICC cases may also be identified and referred for further evaluation, representing an additional clinical benefit beyond the primary screening target. This is particularly relevant given the aggressive nature of ICC and the lack of effective screening tools specifically for this subtype. While we acknowledge that the ICC findings are preliminary and require further validation in larger cohorts, our results suggest that a metabolic signature primarily designed for HCC detection may offer ancillary value for ICC detection, thereby enhancing the overall yield of screening programs.

Several limitations of our study should be acknowledged. First, the cases were recruited from a hospital‐based population, which may introduce spectrum bias and potentially overestimate the model's performance when applied to a general screening context. Future validation in an asymptomatic, population‐based cohort is therefore essential.  Of note, we have filed a patent application for the 9‐M panel combined with AFP, and prospective validation in established LC screening cohorts [6] is currently underway. Second, in the large cohort (n = 2,149), we include a 5‐standard mixture QC instead of pooled QC prepared from all biological samples. While this supports instrument stability, a pooled QC would have provided a more comprehensive assessment of metabolite‐level reproducibility across the full metabolome. Future large‐scale studies should incorporate pooled QC samples prepared from equal‐volume mixtures of all biological samples to further strengthen data quality assessment. Third, although not all metabolites included in the final panel showed fold changes greater than 1.5, they all passed stringent FDR correction, indicating that their selection was statistically robust despite modest effect sizes. Fourth, while our exploratory analyses suggested potential utility of the 9‐M+AFP panel for ICC detection, the limited number of ICC cases, particularly in the external validation cohort, precludes robust conclusions regarding the model's diagnostic performance for this subtype. Moreover, the absence of mechanistic experiments in ICC cell lines means that the functional role of NAM and the biological relevance of the metabolic signature in ICC remain unexplored. Future studies with larger ICC cohorts and dedicated ICC models are warranted to validate both the diagnostic and mechanistic findings for this subtype.

In summary, we developed a 9‐metabolite signature combined with AFP that showed strong discriminatory performance for LC, including early‐stage disease, in a multicenter hospital‐based cohort and an independent external validation cohort. The model performed particularly well for HCC, whereas its performance for ICC remains exploratory because of the limited number of ICC cases. NAM was associated with HBV‐related LC and promoted LC cell proliferation, migration, and invasion in vitro, potentially through an NAD^+^/SIRT1‐dependent HIF1α pathway. These findings support further prospective validation and mechanistic investigation, particularly in population‐based surveillance cohorts and ICC models.

## 4 Experimental Section/Methods

### 4.1 Study Design and Participant Recruitment

This was a multicenter, two‐phase study comprising a retrospective discovery phase and an external validation phase. LC diagnosis was confirmed by histopathological examination. Exclusion criteria included any history of prior cancer other than the newly diagnosed LC, prior surgical treatment, radiotherapy, chemotherapy, or any other targeted therapies. Control subjects were required to have no evidence of LC based on clinical assessment and imaging, with no diagnosis of LC during the relevant follow‐up period, where available. All HBsAg‐positive individuals enrolled in this study had a duration of HBV infection exceeding 6 months and were clinically diagnosed with CHB.

The discovery set consisted of serum samples from 11 clinical centers across five Chinese provinces (Beijing, Inner Mongolia, Hebei, Henan, and Guangdong). Between January 2017 and December 2023, a total of 2,088 participants were retrospectively identified and included. After excluding 164 individuals aged < 35 years, 1,924 eligible participants (1,013 LC patients and 911 non‐LC controls) were included for model development and optimization.

The external validation set comprised a completely independent cohort from Shanxi Cancer Hospital and Hohhot Second Hospital. From January 2024 to July 2024, 245 individuals were enrolled. Following the exclusion of 20 participants under 35 years of age, 225 subjects (100 LC patients and 125 non‐LC controls) were included for external validation of the model. Detailed participant flow and demographic characteristics are presented in Figure 1 and Table 1.

This multicenter study was reviewed and approved by the Institutional Review Board (IRB) of the National Cancer Center/National Clinical Research Center for Cancer/Cancer Hospital, Chinese Academy of Medical Sciences, and Peking Union Medical College, which served as the central IRB and assumed overall ethical responsibility for all participating centers (Approval No. 24/046‐4326).

### 4.2 Serum Sample Collection

Serum samples used in this study were retrieved from archived biospecimens that had been collected and stored at each participating center. All samples had been originally obtained from peripheral venous blood of LC patients and control subjects. For LC patients, blood had been drawn prior to any surgical intervention, whereas control samples had been collected during routine outpatient visits. The original collection procedure involved drawing approximately 5 mL of peripheral venous blood into yellow‐top vacuum tubes containing a clot activator, followed by centrifugation within 2 h to separate the serum. The separated serum was then aliquoted into labeled microcentrifuge tubes and stored at −80°C until use. Before testing, all samples were transferred to the National Cancer Center Biobank for sample information verification.

### 4.3 Quantification of Serum AFP

Serum AFP concentrations were quantified in a batch analysis following sample collection to minimize inter‐assay variability. The concentration of serum AFP was measured using a standardized chemiluminescence immunoassay. The analysis was performed on a Gi2000 automated immunoassay system utilizing a specific reagent kit (Beijing Strong Biotechnology Co., Ltd., China; Catalog number: AFP100L). All procedures were carried out in strict accordance with the manufacturer's protocol. AFP was dichotomized at the cutoff of 20 ng/mL (AFP < 20 ng/mL vs. AFP ≥ 20 ng/mL). This binary classification strategy was applied for AFP diagnostic analysis.

### 4.4 NPELDI‐MS Analysis

Fe_3_O_4_ nanoparticles were synthesized via a hydrothermal method to enhance ionization efficiency in mass spectrometry analysis. Briefly, 0.6 g of trisodium citrate dihydrate and 2.4 g of FeCl_3_·6H_2_O were dissolved in 80 mL of ethylene glycol under sonication for 100 min, yielding a homogeneous orange solution. Thereafter, 3.84 g of anhydrous sodium acetate was introduced into the mixture, followed by an additional 45 min of sonication. The dissolved solutions were transferred to a 200 mL Teflon‐lined stainless‐steel hydrothermal autoclave and subjected to hydrothermal treatment at 200 °C for 10 h in a drying oven. The system was then allowed to cool naturally to room temperature over a period of 10 h. Finally, the obtained sediments were collected, washed sequentially with deionized water and anhydrous ethanol (twice each), and dried at 60 °C for 24 h. All personnel involved in experimental work were blinded to the identities of samples. Five metabolites (valine, aspartic acid, lysine, glucose, and sucrose) with a wide range of molecular weights and polarities were dissolved in deionized water at a concentration of 1 mg/mL and mixed in equal proportions to form the QC sample. We detected one QC sample after every 45 real sample tests. A total of 48 QC samples were performed across the analytical sequence.

During the NPELDI‐MS analysis, 1 µL of 10‐fold diluted serum was deposited onto the target plate and air‐dried. Subsequently, 1 µL of nanoparticle (1 mg/mL dissolved in deionized water) was overlaid onto the pre‐deposited sample and dried at room temperature. NPELDI‐MS analysis was performed in positive‐ion reflector mode with the following optimized parameters: laser intensity at 35% of maximum power, 20 kV acceleration voltage, and 200 Hz pulse repetition rate. All acquired mass spectra were exported in raw data format for subsequent processing.

### 4.5 Non‐Targeted Metabolomics Profiling

PyCharm Community (edition 2022.3) was utilized to analyze the mass spectrometric data. Spectral preprocessing included baseline correction and Savitzky‐Golay smoothing to reduce noise artifacts. Only peaks demonstrating both signal to noise ratios exceeding 3.0 and presence in two‐thirds of replicates were retained as metabolic features. The metabolites were structurally verified by Fourier‐transform ion cyclotron resonance mass spectrometry (FT‐ICR MS) prior to biological identification through Human Metabolome Database (https://hmdb.ca/) matching. The preliminary annotation of the differential metabolites was achieved at Level 3 identification. However, to ensure the reliability of our core findings, the 11 key metabolites in the detection model were further validated using authentic analytical standards. By comparing and matching their mass spectrometric characteristics with these standards, the identification of these 11 metabolites was elevated to Level 2. t‐SNE, PCA, and UMAP were performed on Z‐score‐normalized data by Hiplot (https://hiplot.com.cn/) to visualize the metabolic profiling within and between groups of LC and non‐LC.

### 4.6 Identification and Validation of Differential Metabolites

Raw serum metabolite peak intensities were first normalized to correct inter‐sample technical variation, followed by log‐transformation to alleviate the skewed distribution of metabolomic data. In the internal analysis, preliminary screening of differential metabolites was first performed based on the results of normality tests, using either a two‐tailed t‐test or Wilcoxon rank‐sum test to calculate p‐values. To avoid multicollinearity, multiple comparison corrections were applied to the p‐values using the Benjamini‐Hochberg (BH) method to calculate the FDR value (P.adjusted) [8]. Further analysis using orthogonal partial least squares discriminant analysis (OPLS‐DA) was conducted to demonstrate the differences between the case group and control group [8]. Subsequently, Lasso regression was performed to calculate the importance scores of all metabolic features, which were then ranked in descending order. Notably, LASSO regression was used solely for feature selection and dimension reduction to exclude redundant metabolic features, rather than for building the final diagnostic model. First, duplicated features and those corresponding to unknown metabolites were excluded, and further filtering was conducted by removing features with LASSO regression scores less than 0.20. Then, we optimized the number of selected metabolic features to enhance the model's AUC. The Naïve Bayes classifier was ultimately chosen as the final diagnostic model owing to its favorable predictive performance, simple structure, and high interpretability. To ensure robustness and mitigate overfitting, a Naïve Bayes classifier was trained using this panel and evaluated via 5‐fold cross‐validation in the discovery set. The model's diagnostic performance was assessed based on sensitivity, specificity, and AUC, both in the discovery set and the external validation set.

### 4.7 Development and Validation of the Diagnostic Model

For the development and validation of the LC diagnostic model, the Naïve Bayes algorithm was implemented using the dedicated data mining toolkit Orange [43] (version 3.36.1, the Bioinformatics Lab at the University of Ljubljana, Slovenia). For Lasso regression score (LRScore)‐based feature evaluation, the ‘Rank’ widget in Orange was employed, which was connected to the Lasso regression model to obtain the LRScore for each metabolic feature. All feature selection, model training, and optimization were exclusively performed within the discovery cohort to prevent data leakage. Stratified 5‑fold cross‑validation was implemented for internal model evaluation in the discovery dataset. Upon completion of model training, the fixed model parameters were directly applied to the external validation dataset for blind external testing without any further adjustment. AUC values were computed based on predicted probabilities. For the calculation of classification metrics including sensitivity and specificity, a fixed probability cutoff of 0.5 was used to generate binary class predictions. All other data analyses were performed using R software (v4.4.2, RRID: SCR_001905; R Project for Statistical Computing, http://www.r‐project.org/). The diagnostic task was defined as binary classification, with the Naïve Bayes model outputting a continuous probability score (ranging from 0 to 1) representing the likelihood of cancer presence in each patient [44]. Propensity scores were utilized to adjust for potential confounding factors, including age, gender, and location. Additionally, the diagnostic performance of the metabolite panel was specifically evaluated in subgroups of individuals positive for hepatitis B surface antigen (HBsAg) and negative for AFP.

### 4.8 Associations of the Biomarker Panel With Clinical Characteristics

Pathway enrichment analysis was performed using the Pathway Analysis feature in MetaboAnalyst 6.0 (https://www.metaboanalyst.ca). We collected nine clinical test indicators from 443 subjects in Beijing cohort, including AFP, albumin, alanine aminotransferase (ALT), AST, CHE, globulin, PT, PTA, and total bilirubin (TBIL). After cleaning missing values, clinical information from a total of 256 subjects was included in the study. Spearman correlation analysis was performed between clinical indicators and metabolites, with FDR < 0.05 indicating significant correlation.

### 4.9 Mendelian Randomization Analysis

Data sources were listed in Table S5. Summary‐level statistics of genetic associations with levels of serum NAM were extracted from a comprehensive metabolomic genome‐wide association study (GWAS) in 8,299 individuals of European ancestry (Table S6) [45]. LC association data were obtained from a GWAS involving 947 cases and 378,749 controls from the FinnGen consortium R12, which collected and analyzed genetic and health‐related data from about 500,000 participants. The association analyses in GWAS summary data were performed with adjustment for sex, age, and the first ten principal components. Furthermore, we also utilized data from the “Million Veterans Program”, which included 2,852 cases of LC and 447,587 control samples, all of which were of European ancestry. The details were described in a previous study [46].

The initial investigation of the causal relationship between NAM and the risk of LC used the inverse‐variance weighted (IVW) method, which combines the estimates from each genetic variant to provide a single causal effect estimate, assuming all IVs are valid and there is no horizontal pleiotropy [47]. To test for pleiotropy, we employed MR‐Egger regression, which allows for a non‐zero intercept, thereby indicating the presence of directional pleiotropy [48, 49]. The weighted median was used to obtain consistent estimates even when some of the instruments are invalid, enhancing the reliability of our findings. This approach can effectively detect and eliminate outliers affected by horizontal pleiotropy. Besides, under an assumption of a linear relationship between exposure and outcome, the maximum likelihood‐based method offered a normal bivariate distribution for the estimated causal association [50]. Finally, we conducted random‐effects meta‐analysis to combine MR estimates from different data sources. An observed P‐value < 0.05 was considered statistically significant evidence for a causal association.

### 4.10 Cell Culture and Reagents

Human LC cell lines HepG2 and Hep3B were purchased from American Type Culture Collection (ATCC). HepG2 cells were grown in MEM and Hep3B cells were grown in DMEM, both supplemented with 10% fetal bovine serum (FBS) at 37°C with 5% CO_2_ in a humid atmosphere. NAM (#S1899), Daporinad (#S2799), EX527 (#S1541), and PX‐478 Dihydrochloride (#S7612) were all purchased from Selleck. MG132 (#HY‐13259) was purchased from MCE.

### 4.11 Cell Viability Assay

HepG2 and Hep3B cells were harvested at 80% confluence using 0.25% trypsin, centrifuged, and resuspended to a density of 1‐3 × 10^4^ cells/mL. The cell suspension was seeded into 96‐well plates (100 µL per well) and allowed to adhere for 12–16 h. Subsequently, the cells were treated with a range of concentrations of NAM. Following 72 h of incubation, cell viability was determined by adding 10 µL of CCK‐8 solution to each well. After a further 4 h of incubation, the absorbance was measured at 450 nm using a microplate reader (Multiskan FC, Thermo Fisher Scientific, USA).

### 4.12 Cell Migration and Invasion Assays

Cell migration was assessed using Transwell inserts with an 8 µm pore size. Serum‐starved HepG2 and Hep3B cells were resuspended in serum‐free medium and seeded into the upper chamber. The lower chamber was filled with complete medium supplemented with 10% FBS, which served as a chemoattractant. After 48 h of incubation at 37°C, non‐migratory cells on the upper surface of the membrane were carefully removed with a cotton swab. Cells that had migrated to the lower surface were fixed with 4% paraformaldehyde for 15 min, stained with 0.1% crystal violet for 20 min, rinsed with PBS, and air‐dried. The number of migrated cells was quantified by counting three random microscopic fields per insert. For the invasion assay, the same procedure was followed, with the exception that the Transwell inserts were pre‐coated with Matrigel to simulate the extracellular matrix barrier.

### 4.13 NAD^+^ and NADH Detection Assay

Intracellular NAD^+^ and NADH levels were measured in HepG2 cells (1 × 10^6^ cells per sample) following treatment with NAM, using a commercial NAD^+^/NADH assay kit according to the manufacturer's protocol (Beyotime). Briefly, cells were lysed with 200 µL of ice‐cold lysis buffer. For total NAD^+^/NADH measurement, 20 µL of lysate was transferred directly to a 96‐well plate. For NADH‐specific measurement, an aliquot of lysate was heated at 60°C for 30 min to decompose NAD^+^ prior to plating. Subsequently, 90 µL of alcohol dehydrogenase working solution was added to each well and incubated at 37°C for 10 min, followed by the addition of 10 µL of chromogenic solution and a further 30 min incubation at 37°C. Absorbance was measured at 450 nm using a microplate reader. NAD^+^ concentration was calculated by subtracting the NADH value from the total NAD^+^/NADH value. A standard curve was run in parallel for quantification.

### 4.14 Immunoprecipitation (IP) and Immunoblotting (IB)

HepG2 cells were harvested and lysed in RIPA buffer (50 mM Tris‐HCl pH 7.4, 1% NP40, 0.5% Na‐deoxycholate, 0.1% SDS, 150 mM NaCl, 2 mM EDTA, and 50 mM NaF) supplemented with protease inhibitor cocktail (Sigma) and Phenylmethylsulfonyl Fluoride (Sigma) on ice for 30 min. Cell lysates were cleared by centrifuging at 12,000 rpm, 4°C for 20 min, and the protein concentration was determined by BCA assay (Beyotime). Equal amounts of protein (20–40 µg) per sample were used in the following IB procedures [51]. For IP, primary antibodies were incubated with lysates at 4°C overnight. Protein G agaroses were then added, and the incubation was continued for an additional 2 h at 4°C. Agarose beads were then washed with lysis buffer three times, and the immunoprecipitates were eluted and analyzed by IB. The antibodies used were anti‐HIF1α (#20960‐1‐AP, Proteintech; 1:2000 dilution), anti‐Glut1 (#21829‐1‐AP, Proteintech; 1:1000 dilution), anti‐VEGF (#66828‐1‐Ig, Proteintech; 1:1000 dilution), anti‐phospho‐ERK1/2 (#4370, CST; 1:1000 dilution), anti‐ERK1/2 (#4695, CST; 1:1000 dilution), anti‐Acetyl‐p53K382 (#ab75754, abcam; 1:500 dilution), anti‐pan‐acetyl (#PTM‐105RM, PTMBIO; 1:500 dilution), anti‐SIRT1 (#ab189494, abcam; 1:1000 dilution), anti‐ubiquitin (#10201‐2‐AP, Proteintech; 1:5000 dilution) and anti‐β‐actin (#66009‐1‐Ig, 1:10000 dilution).

### 4.15 Lactate Detection Assay

The L‐lactate concentration in HepG2 cell culture supernatants was quantified using a commercial L‐Lactic Acid Colorimetric Assay Kit (Elabscience, E‐BC‐K044‐M). Briefly, cell supernatants were collected and used directly without dilution. For the assay, 5 µL of supernatant was added to the microplate well, followed by 100 µL of freshly prepared enzyme working solution (a mixture of Buffer and Enzyme Stock Solution at a 10:1 ratio) and 20 µL of Chromogenic Agent. The reaction mixture was incubated at 37°C for 10 min. The reaction was then stopped by adding 180 µL of Stop Solution. After mixing for 5 s, the absorbance was measured at 530 nm using a microplate reader. The L‐lactate concentration (mmol/L) in the samples was calculated based on a standard curve generated from known concentrations of L‐lactic acid standard.

### 4.16 Data Analysis

The sample size was estimated based on the anticipated AUC and the desired precision of the 95% CI. The calculation assumed an expected AUC of 0.80 and a margin of error of 0.10 at a two‐sided α level of 0.05. This calculation was intended for the primary LC –vs.‐control analysis and  at least 62 LC cases and 62 controls are required to ensure statistical power [52]. Therefore, the sample sizes of the discovery dataset and external validation dataset in this study meet the necessary methodological requirements.

Normality of continuous variables was evaluated using the Shapiro–Wilk test. Normally distributed continuous data were summarized as mean ± standard deviation (SD) and compared between groups via t‐tests, whereas non‐normally distributed data were reported as median (25th percentile, 75th percentile) and compared between groups via Wilcoxon rank‐sum tests. In this study, both age and AFP exhibited non‐normal distributions. Categorical variables were presented as counts (percentages). Intergroup differences were assessed using the chi‐square (χ^2^) test; Fisher's exact test was utilized when any cell had an observed frequency below 5. The 95% CI of the AUC was calculated using the DeLong method, and the 95% CIs for sensitivity and specificity at a fixed cutoff of 0.5 were computed using the Wilson score interval. All statistical tests were two‐tailed, and a P value < 0.05 was defined as the threshold for statistical significance. All analyses were performed using R v4.4.2.

## Author Contributions

**Yongjie Xu**: conceptualization, methodology, formal analysis, project administration, writing – original draft, writing – review and editing, software, visualization. **Jiachen Wang**: writing – original draft, writing – review and editing, methodology, software, conceptualization, formal analysis, visualization. **Chunmeng Ding**: conceptualization, methodology, software, writing – original draft, writing – review and editing, formal analysis, visualization. **Changfa Xia**: conceptualization, methodology, supervision, investigation, validation. **Yunyong Liu**: conceptualization, supervision, validation, investigation. **Yuanjie Zheng**: data curation, validation, investigation. **Sheng Chang**: funding acquisition, supervision. **Shaokai Zhang**: resources, supervision, investigation. **Yutong He**: investigation, supervision, resources. **Ruifang Sun**: resources, supervision, investigation. **Yunfeng Xi**: validation, supervision, resources. **Yan Liu**: data curation, resources. **Kun Qian**: writing – review and editing, conceptualization, methodology, supervision, project administration. **Wanqing Chen**: writing – review and editing, conceptualization, funding acquisition, supervision, project administration.

## Conflicts of Interest

The authors declare no conflicts of interest.

## Supporting information




**Supporting File**: advs78077‐sup‐0001‐SuppMat.docx.

## Data Availability

The data that support the findings of this study are available on request from the corresponding author. The data are not publicly available due to privacy or ethical restrictions.
