# Supplementary material for: Metabolic Signatures for Liver Cancer Diagnosis and Mechanistic Insights: A Large‐Scale, Multicenter Study
Source: Adv Sci (Weinh). 2026 Oct 4:e78077. Online ahead of print. doi: 10.1002/advs.78077 (PMC13634481; doi:10.1002/advs.78077)
Supplement: Supplementary file 1 [file ADVS-9999-e78077-s001.docx]

Supporting Information

**Metabolic signatures for liver cancer diagnosis and mechanistic insights: a large-scale, multicenter study**

*Yongjie Xu^#^, Jiachen Wang^#^, Chunmeng Ding^#^, Changfa Xia^#^, Yunyong Liu^#^, Yuanjie Zheng, Sheng Chang, Shaokai Zhang, Yutong He, Ruifang Sun, Yunfeng Xi, Yan Liu*, Kun Qian*, Wanqing Chen**

**List of Supplementary Materials**

Figure S1 to S6

Table S1 to S6

**Supplementary figures**

**Figure S1. Distribution of serum AFP levels in patients with LC and non-LC in the internal and external validation cohorts.** Boxplots illustrate the interquartile range (IQR), with the horizontal line inside each box representing the median AFP concentration. Whiskers extend to the furthest observations within 1.5×IQR, and individual points denote outliers beyond this range. The y-axis is presented on a log_10_ scale to accommodate the skewed distribution of AFP. The grey dashed horizontal line indicates the AFP cutoff value of 20 ng/mL. After excluding participants with missing data, the sample sizes were as follows: internal non-LC = 904, internal LC = 994, external non-LC = 125, external LC = 100. Median AFP values were markedly higher in LC patients than non-LC individuals in both internal (8.06 vs. 1.71, p < 0.001) and external cohorts (11.01 vs. 1.09, p < 0.001). LC, liver cancer.

**Figure S2. Stability of the metabolic detection.** (**A**) PCA score plot of all 48 QC samples. (**B**) Intensity scatterplot and RSD bar for five metabolite detections in all of the quality control samples. PCA, principal component analysis. QC, quality control. RSD, relative standard deviation.

**Figure S3. Comparison of the relative abundance of 11 metabolites between non-LC and LC groups.** The raw abundance values of 11 metabolites were used for statistical analysis, while log10-transformed values were applied for visualization. Differences in metabolite levels between the non-LC and LC groups were evaluated using Wilcoxon rank-sum test according to the normality of data distribution. Blue boxes represent the non-LC group and red boxes represent the LC group. LC, liver cancer. **p<0.01, ***p<0.001.

**Figure S4. Construction and validation of LC diagnostic models.** (**A**) ROC curve of 9-M+AFP Naïve Bayes model in distinguishing LC from cirrhosis. **(B)** ROC curve of 9-M+AFP Naïve Bayes model in the diagnosis of AFP-negative early-stage LC (Stage I & II). (**C**) DCA of three predictive models for LC. Net benefit was plotted against threshold probability. The red line represents the combined model (9M+AFP); The gray line “All” indicates the strategy of treating all patients, and the black line “None” represents treating no patients. DCA, decision curve analysis. LC, liver cancer. ROC, receiver operating characteristic.

**Figure S5. NAM promotes LC cell proliferation, migration, and invasion in an NAD^+^/SIRT1-dependent pathway. (A)** Scatterplot illustrating Nicotinamide in the main analysis (inverse-variance weighted, IVW method) and several sensitivity analyses in Figgen data (left) and VA Million Veteran Program (MVP) data (right). Data were presented as 95% CI. **(B)** Cell viability of Hep3B cells treated with the indicated concentrations of NAM for 72 h. (**C**) Representative images and quantification of migration and invasion assays for Hep3B cells treated with the indicated concentrations of NAM. Scale bar, 200 μm. (**D**) mRNA expression levels of SLC2A1 (encoding Glut1) and VEGFA (encoding VEGF) in Hep3B cells treated with increasing concentrations of NAM. (**E**) Western blot analysis of protein expression in Hep3B cells following treatment with different concentrations of NAM. (**F**) Intracellular LA production in Hep3B cells after treatment with various concentrations of NAM. NAM, nicotinamide. LA, lactate. LC, liver cancer**.** *p<0.05, **p<0.01, ***p<0.001, ****p<0.0001. n.s., not significant.

**Figure S6. NAM upregulates HIF1α expression via an NAD^+^/SIRT1-dependent pathway. (A)** Intracellular NAD⁺ levels and NAD⁺/NADH ratio in HepG2 cells treated with 200 µM NAM for 12 h or 24 h. (B) CHX chase assay. HepG2 cells were treated with 10 μg/mL CHX for the indicated time points, with or without 50 μM EX527 pretreatment. HIF1α protein levels were assessed by immunoblotting. Quantification of HIF1α protein decay curves is shown. Data are presented as mean ± SD from three independent experiments. CHX, cycloheximide. NAM, nicotinamide. *p < 0.05, **p < 0.01.

**Supplementary Tables**

**Table S1.** Annotation of the 37 differential metabolic features.

| **FT_MEASURED_m/z** | **POTENTIAL BIOCHEMICAL** | **FORMULA** | **HMDB ID** | **THEORATICAL_m/z** | **ADDUCT** | **PPM** |
| --- | --- | --- | --- | --- | --- | --- |
| 112.0369 | L-alanine | C3H7NO2 | HMDB0000161 | 112.0369 | M+Na | 0.01 |
| 118.0264 | 2-Hydroxypyridine | C5H5NO | HMDB0013751 | 118.0263 | M+Na | 0.56 |
| 118.9717 | Glyoxylic acid | C2H2O3 | HMDB0000119 | 118.9716 | M+2Na-H | 1.18 |
| 123.0552 | Nicotinamide | C6H6N2O | HMDB0001406 | 123.0553 | M+H | -0.73 |
| 124.0381 | Nicotinic acid | C6H5NO2 | HMDB0001488 | 124.0393 | M+H | -9.72 |
| 125.021 | Acetoacetic acid | C4H6O3 | HMDB0000060 | 125.0209 | M+Na | 0.68 |
| 141.0524 | 2-Hydroxy-3-methylbutyric acid | C5H10O3 | HMDB0000407 | 141.0522 | M+Na | 1.31 |
| 141.6419 | NA | NA | NA | NA | NA | NA |
| 141.5355 | NA | NA | NA | NA | NA | NA |
| 142.948 | Phosphate | H3O4P | HMDB0001429 | 142.9481 | M+2Na-H | -0.43 |
| 146.0204 | Nicotinic acid | C6H5NO2 | HMDB0001488 | 146.0212 | M+Na | -5.82 |
| 185.9541 | Carbamoyl phosphate | CH4NO5P | HMDB0001096 | 185.9539 | M+2Na-H | 1.21 |
| 188.0299 | Vinylacetylglycine | C6H9NO3 | HMDB0000894 | 188.0294 | M+2Na-H | 2.61 |
| 201.9339 | Taurine | C2H7NO3S | HMDB0000251 | 201.9337 | M+2K-H | 0.96 |
| 205.06844 | Galactitol | C6H14O6 | HMDB0000107 | 205.0683 | M+Na | 0.88 |
| 206.0193 | Indole-3-carboxylic acid | C9H7NO2 | HMDB0003320 | 206.0188 | M+2Na-H | 2.22 |
| 210.0498 | Phenylalanine | C9H11NO2 | HMDB0000159 | 210.0501 | M+2Na-H | -1.63 |
| 226.0453 | L-Tyrosine | C9H11NO3 | HMDB0000158 | 226.0451 | M+2Na-H | 1.07 |
| 227.0794 | L-Tryptophan | C11H12N2O2 | HMDB0000929 | 227.0791 | M+Na | 1.33 |
| 230.0438 | 5-Phosphoribosylamine | C5H12NO7P | HMDB0001128 | 230.0424 | M+H | 6.01 |
| 241.9984 | Phenylalanine | C9H11NO2 | HMDB0000159 | 241.9980 | M+2K-H | 1.57 |
| 242.9433 | Phthalic acid | C8H6O4 | HMDB0002107 | 242.9456 | M+2K-H | -9.47 |
| 257.9904 | L-Tyrosine | C9H11NO3 | HMDB0000158 | 257.9929 | M+2K-H | -9.82 |
| 258.963 | 1-Methyluric acid | C6H6N4O3 | HMDB0003099 | 258.9630 | M+2K-H | -0.12 |
| 282.8825 | NA | NA | NA | NA | NA | NA |
| 303.2301 | Linoleic acid | C18H32O2 | HMDB0000673 | 303.2295 | M+Na | 2.14 |
| 304.9808 | 5-Methylthioribose 1-phosphate | C6H13O7PS | HMDB0000963 | 304.9831 | M+2Na-H | -7.63 |
| 320.9915 | Uridine | C9H12N2O6 | HMDB0000296 | 320.9886 | M+2K-H | 9.10 |
| 409.3434 | Cholesterol | C27H46O | HMDB0000067 | 409.3441 | M+Na | -1.67 |
| 410.2668 | N-Stearoyl Cysteine | C21H41NO3S | HMDB0241940 | 410.2699 | M+Na | -7.64 |
| 429.1828 | MG (18:3(9Z,12Z,15Z)/0:0/0:0) | C21H36O4 | HMDB0011570 | 429.1804 | M+2K-H | 5.59 |
| 449.2472 | MG (0:0/PGD2/0:0) | C23H38O7 | HMDB0260528 | 449.2510 | M+Na | -8.46 |
| 450.3231 | Deoxycholic acid glycine conjugate | C26H43NO5 | HMDB0000631 | 450.3214 | M+H | 3.77 |
| 451.2404 | 7-Ketodeoxycholic acid | C24H38O5 | HMDB0000391 | 451.2431 | M+2Na-H | -5.96 |
| 465.25024 | Dehydroepiandrosterone 3-glucuronide | C25H36O8 | HMDB0010327 | 465.2483 | M+H | 4.18 |
| 466.1467 | Dihydrofolic acid | C19H21N7O6 | HMDB0001056 | 466.1446 | M+Na | 4.60 |
| 467.3303 | Cholesteryl acetate | C29H48O2 | HMDB0003822 | 467.3286 | M+K | 3.66 |

FT_MEASURED_m/z, m/z of signals mearsured by Fourier transform-ion cyclotron resonance-mass spectrometry (FT-ICR-MS).

**Table S2.** Dysregulated metabolites in liver cancer.

| **Metabolites** | **Log**_2_FC | **FDR** | **Regulation** | **LASSO coefficients** |
| --- | --- | --- | --- | --- |
| 2-Hydroxy-3-methylbutyric acid | -0.09 | 0.002 | Down | 1.48 |
| L-Tyrosine | 0.14 | 0.004 | Up | 1.10 |
| Carbamoyl phosphate | -0.17 | 0.001 | Down | 0.93 |
| Nicotinamide | 0.09 | 0.01 | Up | 0.92 |
| Phenylalanine | 0.36 | <0.001 | Down | 0.61 |
| Glyoxylic acid | 0.1 | <0.001 | Up | 0.55 |
| Uridine | -0.33 | 0.02 | Down | 0.54 |
| Acetoacetic acid | -0.05 | 0.05 | Down | 0.30 |
| Linoleic acid | -0.16 | 0.01 | Down | 0.26 |
| L-alanine | -0.08 | 0.03 | Down | 0.25 |
| 1-Methyluric acid | -0.42 | 0.006 | Down | 0.20 |
| 5-Methylthioribose 1-phosphate | -0.17 | 0.01 | Down | 0.17 |
| Dihydrofolic acid | 0.32 | <0.001 | Up | 0.16 |
| MG (18:3(9Z,12Z,15Z)/0:0/0:0) | 0.14 | <0.001 | Up | 0.11 |
| Cholesteryl acetate | 0.24 | <0.001 | Up | 0.07 |
| Vinylacetylglycine | 0.11 | 0.03 | Up | 0.06 |
| Dehydroepiandrosterone 3-glucuronide | 0.33 | <0.001 | Up | 0.03 |
| Taurine | -0.2 | 0.003 | Down | 0.03 |
| Indole-3-carboxylic acid | -0.1 | 0.02 | Down | 0.03 |
| Cholesterol | -0.07 | 0.03 | Down | 0.03 |
| L-Tryptophan | 0.25 | 0.01 | Up | 0.02 |
| 7-Ketodeoxycholic acid | 0.12 | 0.003 | Up | 0.02 |
| Nicotinic acid | 0.25 | <0.001 | Up | 0.02 |
| 5-Phosphoribosylamine | -0.08 | 0.01 | Down | 0.00 |
| 2-Hydroxypyridine | 0.11 | 0.02 | Up | 0.00 |
| Galactitol | 0.19 | 0.004 | Up | 0.00 |
| Phosphate | 0.14 | 0.02 | Up | 0.00 |
| Phthalic acid | -0.23 | 0.003 | Down | 0.00 |
| N-Stearoyl Cysteine | -0.07 | 0.02 | Down | 0.00 |
| MG (0:0/PGD2/0:0) | 0.19 | <0.001 | Up | 0.00 |
| Deoxycholic acid glycine conjugate | 0.18 | <0.001 | Up | 0.00 |

**Table S3.** Diagnostic performance of AFP, the 9-M, and 9-M+AFP across different clinical scenarios.

|  |  | **Discovery set** | | |  | **Validation set** | | |
| --- | --- | --- | --- | --- | --- | --- | --- | --- |
|  |  | AFP | 9-M | 9-M+AFP |  | AFP | 9-M | 9-M+AFP |
| LC versus non-LC | AUC | 0.68  (0.67-0.70) | 0.85  (0.83-0.86) | 0.92  (0.91-0.93) |  | 0.72  (0.67-0.77) | 0.84  (0.79-0.89) | 0.93  (0.90-0.96) |
|  | Sensitivity | 0.39  (0.36-0.42) | 0.76  (0.73-0.79) | 0.84  (0.82-0.86) |  | 0.43  (0.34-0.53) | 0.90  (0.83-0.94) | 0.83  (0.74-0.89) |
|  | Specificity | 0.98  (0.97-0.98) | 0.73  (0.70-0.76) | 0.83  (0.80-0.85) |  | 0.99  (0.96-1.00) | 0.58  (0.50-0.67) | 0.85  (0.77-0.90) |
| Early LC versus  non-LC | AUC | 0.62  (0.59-0.64) | 0.87  (0.84-0.89) | 0.92  (0.90-0.93) |  | 0.72  (0.66-0.79) | 0.85  (0.79-0.91) | 0.93  (0.90-0.96) |
|  | Sensitivity | 0.26  (0.22-0.31) | 0.80  (0.75-0.84) | 0.85  (0.82-0.88) |  | 0.46 (0.34-0.58) | 0.95  (0.86-0.98) | 0.78  (0.66-0.87) |
|  | Specificity | 0.98  (0.97-0.98) | 0.74  (0.71-0.77) | 0.84  (0.82-0.86) |  | 0.99 (0.96-1.00) | 0.58  (0.50-0.67) | 0.85  (0.77-0.90) |

The diagnostic cutoff value of AFP was 20 ng/mL.

**Table S4**. Genetic variants associated with the circulating level of nicotinamide in Mendelian randomization analyses.

|  |  | **SNPs** | **Beta** | **SE** | **OR** | **95%Cl** | ***P*** | **Qrs/Interrupt** |
| --- | --- | --- | --- | --- | --- | --- | --- | --- |
| **VA Million Veteran Program** |  |  |  |  |  |  |  |  |
|  | Inverse-variance weighted (random effects) | 17 | 0.174 | 0.098 | 1.190 | 0.982-1.443 | 0.076 | 0.868 |
|  | Inverse-variance weighted (fixed effects) | 17 | 0.174 | 0.098 | 1.190 | 0.982-1.443 | 0.076 | 0.868 |
|  | MR-Egger | 17 | 0.024 | 0.261 | 1.025 | 0.615-1.708 | 0.926 | 0.845 |
|  | Weighted median | 17 | 0.244 | 0.133 | 0.067 | 0.983-1.657 | 0.067 | NA |
|  | Simple median | 17 | 0.243 | 0.134 | 0.069 | 0.981-1.657 | 0.069 | NA |
|  | Maximum-likelihood method | 17 | 0.183 | 0.100 | 0.067 | 0.987-1.461 | 0.067 | NA |
| **Finngen** |  |  |  |  |  |  |  |  |
|  | Inverse-variance weighted (random effects) | 17 | 0.437 | 0.152 | 1.548 | 1.149-2.086 | 0.004 | 0.360 |
|  | Inverse-variance weighted (fixed effects) | 17 | 0.437 | 0.146 | 1.548 | 1.163-2.060 | 0.003 | 0.360 |
|  | MR-Egger | 17 | 0.388 | 0.345 | 1.473 | 0.750-2.896 | 0.279 | 0.874 |
|  | Weighted median | 17 | 0.546 | 0.220 | 1.726 | 1.122-2.654 | 0.013 | NA |
|  | Simple median | 17 | 0.561 | 0.223 | 1.752 | 1.133-2.710 | 0.012 | NA |
|  | Maximum-likelihood method | 17 | 0.446 | 0.152 | 1.562 | 1.161-2.103 | 0.003 | NA |
| **Meta-Analysis** |  |  |  |  |  |  |  |  |
|  | Inverse-variance weighted | 17 | 0.283 | 0.130 | 1.327 | 1.029-1.712 | 0.029 | NA |

**Table S5**. Details of the genome-wide association studies and datasets used in Mendelian randomization analyses.

| **Exposure or outcome** | **Sample size** | **Ancestry** | **Links for data download** | **PMID** |
| --- | --- | --- | --- | --- |
| 1,400 blood metabolites | 7,824 participants | European ancestry | https://www.ebi.ac.uk/gwas/publications/36635386https://www.ebi.ac.uk/gwas/publications/36635386 | 36635386 |
| Liver cancer | 2,852 cases, 447,587 controls | European ancestry (VA Million Veteran Program) | http://ftp.ebi.ac.uk/pub/databases/gwas/summary_statistics/GCST90475001-GCST90476000/GCST90475569/http://ftp.ebi.ac.uk/pub/databases/gwas/summary_statistics/GCST90475001-GCST90476000/GCST90475569/ | 39024449 |
|  | 947 cases, 378,749 controls | European ancestry (Finngen R12) | https://storage.googleapis.com/finngen-public-data-r12/summary_stats/release/finngen_R12_C3_HEPATOCELLU_CARC_EXALLC.gzhttps://storage.googleapis.com/finngen-public-data-r12/summary_stats/release/finngen_R12_C3_HEPATOCELLU_CARC_EXALLC.gz | 36653562 |

**Table S6**. Characteristics of the genetic variants associated with the circulating level of nicotinamide.

| SNP | chr | pos | Effect allele | Other allele | beta | se | pval |
| --- | --- | --- | --- | --- | --- | --- | --- |
| rs148244996 | 1 | 197738335 | T | A | 0.378517 | 0.0841454 | 6.85E-06 |
| rs67524001 | 2 | 235161263 | T | C | 0.0807149 | 0.0166249 | 1.20E-06 |
| rs114328071 | 3 | 112129254 | A | G | -0.255296 | 0.0551812 | 3.72E-06 |
| rs4132577 | 3 | 95172463 | G | C | 0.0722496 | 0.0154464 | 2.90E-06 |
| rs6798308 | 3 | 162177521 | C | T | -0.0705107 | 0.015621 | 6.37E-06 |
| rs6835705 | 4 | 15736747 | A | G | 0.197354 | 0.0432485 | 5.04E-06 |
| rs7657217 | 4 | 168840554 | T | C | -0.0997855 | 0.0218101 | 4.76E-06 |
| rs6887037 | 5 | 174792086 | A | T | 0.079635 | 0.0160282 | 6.75E-07 |
| rs722585 | 6 | 1775629 | A | G | 0.0804135 | 0.0171178 | 2.63E-06 |
| rs9373605 | 6 | 101211446 | A | G | 0.122538 | 0.0249828 | 9.35E-07 |
| rs579090 | 7 | 54728759 | A | T | -0.233213 | 0.0516028 | 6.20E-06 |
| rs62521592 | 8 | 125621287 | T | C | -0.149501 | 0.0334328 | 7.76E-06 |
| rs72686880 | 9 | 18390839 | C | T | -0.0972767 | 0.0211281 | 4.14E-06 |
| rs4888987 | 16 | 79485932 | C | G | -0.102569 | 0.0224792 | 5.05E-06 |
| rs72795906 | 16 | 29002728 | A | G | 0.0922294 | 0.0206275 | 7.78E-06 |
| rs145168145 | 19 | 39367913 | T | G | 0.413821 | 0.093591 | 9.80E-06 |
| rs56234300 | 20 | 41396613 | G | T | 0.171361 | 0.0373122 | 4.38E-06 |
